# Defense-related transcription factors WRKY70 and WRKY54 modulate osmotic stress tolerance by regulating stomatal aperture in *Arabidopsis*

**DOI:** 10.1111/nph.12378

**Published:** 2013-07-01

**Authors:** Jing Li, Sebastien Besseau, Petri Törönen, Nina Sipari, Hannes Kollist, Liisa Holm, E Tapio Palva

**Affiliations:** 1 Viikki Biocenter, Division of Genetics, Department of Biosciences, University of HelsinkiFI-00014, Helsinki, Finland; 2 Université François Rabelais de ToursEA2106 Biomolécules et Biotechnologies Végétales, 37200, Tours, France; 3 Institute of Biotechnology, University of HelsinkiFI-00014, Helsinki, Finland; 4 Institute of Technology, University of TartuNooruse 1, Tartu, 50411, Estonia

**Keywords:** abscisic acid (ABA), *Arabidopsis*, gene regulation, osmotic stress, salicylic acid (SA), stomata, WRKY transcription factor

## Abstract

- WRKY transcription factors (TFs) have been mainly associated with plant defense, but recent studies have suggested additional roles in the regulation of other physiological processes. Here, we explored the possible contribution of two related group III WRKY TFs, WRKY70 and WRKY54, to osmotic stress tolerance. These TFs are positive regulators of plant defense, and co-operate as negative regulators of salicylic acid (SA) biosynthesis and senescence.
- We employed single and double mutants of *wrky54* and *wrky70*, as well as a *WRKY70* overexpressor line, to explore the role of these TFs in osmotic stress (polyethylene glycol) responses. Their effect on gene expression was characterized by microarrays and verified by quantitative PCR. Stomatal phenotypes were assessed by water retention and stomatal conductance measurements.
- The *wrky54wrky70* double mutants exhibited clearly enhanced tolerance to osmotic stress. However, gene expression analysis showed reduced induction of osmotic stress-responsive genes in addition to reduced accumulation of the osmoprotectant proline. By contrast, the enhanced tolerance was correlated with improved water retention and enhanced stomatal closure.
- These findings demonstrate that WRKY70 and WRKY54 co-operate as negative regulators of stomatal closure and, consequently, osmotic stress tolerance in *Arabidopsis*, suggesting that they have an important role, not only in plant defense, but also in abiotic stress signaling.

## Introduction

In their natural environment, plants are confronted with a series of biotic and abiotic stresses that detrimentally affect their growth and development. Among these, osmotic stress, which results in cellular water deficit, is one of the most limiting factors of plant growth, distribution and crop productivity, and consequently poses a serious threat to the agricultural industry worldwide (Rabbani *et al.*, 2003). The disruption of plant water status and low water potential can be caused by a number of factors, such as decreased water availability in the soil during drought, reduced water uptake as a result of high salinity or freeze-induced cellular dehydration (Verslues *et al.*, 2006). To respond to osmotic stress, plants have evolved complex adaptive strategies that help to avoid or tolerate cellular dehydration, allowing plants to grow and complete their life cycles. The first response of a plant is the control of water balance by stomatal movement. At the cellular level, tolerance to osmotic stress includes enhanced expression of stress-responsive genes and metabolic adjustments, resulting in the accumulation of osmolytes, protective solutes and proteins (Xiong *et al.*, 1999; Verslues *et al.*, 2006; Shinozaki & Yamaguchi-Shinozaki, 2007).

The central phytohormone in osmotic stress perception and signaling is abscisic acid (ABA), which has been implicated in both the control of stomatal aperture and the activation of a distinct set of genes associated with the biosynthesis of osmolytes and protective proteins (Mahajan & Tuteja, 2005; Shinozaki & Yamaguchi-Shinozaki, 2007; Acharya & Assmann, 2009; Hao *et al.*, 2011). Recent advances have succeeded in the identification of PYR/PYL/RCAR ABA receptors which interact with type 2C protein phosphatases (PP2Cs), such as ABI1, HAB1 and AIP1 (Leung *et al.*, 1994; Meyer *et al.*, 1994; Saez *et al.*, 2004; Ma *et al.*, 2009; Park *et al.*, 2009; Lim *et al.*, 2012). The binding of ABA to these cytosolic receptors inactivates the inhibition of PP2Cs on downstream signal transduction, allowing protein kinases, such as SnRK2s, to activate ABF/AREB bZIP transcription factors (TFs) (Umezawa *et al.*, 2009; Santiago *et al.*, 2012). These TFs have a pivotal function during osmotic stress for the induction of ABA-responsive genes (Uno *et al.*, 2000; Antoni *et al.*, 2011; Fujita *et al.*, 2013). In guard cells, ABA perception and PP2C sequestration allow SnRK2s and several calcium-dependent protein kinases (CDPKs) to activate NADPH oxidase and anion channels (SLAC1 and SLAH3) for guard cell closure (Joshi-Saha *et al.*, 2011).

The TFs induced or activated by plant perception of environmental cues are central mediators of transcriptional reprograming which leads to plant adaptation (Chen *et al.*, 2002; Nakashima *et al.*, 2009). In addition to ABF/AREB bZIP TFs, members of several other TF families have been found to regulate the expression of ABA-, drought- or cold-responsive genes, including MYB, MYC, NAC and WRKY TFs (Abe *et al.*, 2003; Fujita *et al.*, 2004; Rushton *et al.*, 2012). The WRKY TF family with > 70 members in *Arabidopsis* is one of the central TF groups involved in biotic stress responses (Ulker & Somssich, 2004; Yamasaki *et al.*, 2005). *WRKY* genes are typically induced by pathogens and salicylic acid (SA), and, in turn, control the expression of defense-related genes (Dong *et al.*, 2003; Ulker & Somssich, 2004). WRKYs have also been implicated in various other physiological and developmental programs, including senescence, seed germination and trichome development (Robatzek & Somssich, 2001; Johnson *et al.*, 2002; Seki *et al.*, 2002; Singh *et al.*, 2002; Besseau *et al.*, 2012). Recent studies, especially in *Arabidopsis* and rice, have indicated that some WRKY TFs also play important roles in transcriptional reprograming during abiotic stresses, such as drought, high salinity, cold and osmotic stress (Chen *et al.*, 2012). In this context, WRKYs have been implicated in ABA signaling and the oxidative stress response (Chen *et al.*, 2010; Rushton *et al.*, 2012). For example, *AtWRKY40* can inhibit directly the expression of important ABA-responsive genes and can function as a negative regulator of ABA signaling in seed germination, in a complex interacting network with the antagonists *AtWRKY18* and *AtWRKY60* (Chen *et al.*, 2010; Shang *et al.*, 2010). However, *AtWRKY63* (ABO3) has been shown to regulate seed germination and seedling growth, and appears to be involved in the control of stomatal closure, consequently affecting the drought tolerance of the plant (Ren *et al.*, 2010). This function in the abiotic stress response is highlighted by the capacity of WRKY40 and WRKY63 to bind directly to the promoters of ABA-responsive ABF/AREB TF genes (Ren *et al.*, 2010; Shang *et al.*, 2010).

Two members of *Arabidopsis* WRKY group III, the closely related WRKY54 and WRKY70 TFs, have been demonstrated to be key components in the regulation of biotic stress response networks integrating signals from SA and jasmonic acid (JA) pathways in plant defense and in the control of SA biosynthesis (Li *et al.*, 2004, 2006; Wang *et al.*, 2006). Furthermore, co-operation of WRKY70 and WRKY54 as negative regulators of leaf senescence in *Arabidopsis* has also been demonstrated (Ulker *et al.*, 2007; Besseau *et al.*, 2012). In this study, we explored the possible role of *WRKY54* and *WRKY70* in abiotic stress tolerance, in particular in adaptation to osmotic stress. We found that *wrky54wrky70* double mutant exhibited enhanced tolerance to osmotic stress. We characterized the involvement of these two WRKYs in the regulation of osmotic stress-related genes and elucidated their potential role in osmotic stress adaptation. Our results suggest that *WRKY54* and *WRKY70* co-operate to modulate stomatal movement and osmotic stress-responsive gene expression through both SA-mediated and SA-independent processes, highlighting the complexity of plant responses to environmental cues and the interactions of signaling networks in plant stress responses.

## Materials and Methods

### Plant material and growth conditions

The growth conditions for the plants are the same as those described by Besseau *et al*. (2012). The plants were grown for 3 or 4 wk before treatments.

The backgrounds of the *Arabidopsis thaliana* (L.) Heynh plants and mutants used were Columbia (Col-0) and Landsberg erecta (Ler) ecotypes. T-DNA mutant lines *wrky54* (SALK_111964) and *wrky70* (SALK_025198) were supplied by the Nottingham Arabidopsis Stock Centre (NASC), Nottingham, UK. Single-mutant characterization and double-mutant production have been described previously (Besseau *et al.*, 2012). The *sid2-1* mutant was provided by J. P. Metraux (University of Fribourg, Switzerland) and was crossed with the *wrky54wrky70* double mutant to obtain the triple mutant *wrky54wrky70sid2-1*. The *abi1-1* mutation was also introduced to the *wrky54wrky70* double mutant to generate the *wrky54wrky70abi1-1* triple mutant. The transgenic line expressing *WRKY70* was produced as described previously (Li *et al.*, 2004).

### Exposure to abiotic stresses and exogenous SA or ABA

Depending on the experiments, two methods were used to induce osmotic stress in plants. Three-week-old plants were watered with 15% polyethylene glycol (PEG)6000 solution during 1–3 d. Plants watered with water were used as a control. Alternatively, 3-wk-old seedlings grown on half-strength Murashige and Skoog (MS) solid medium were transferred to half-strength MS solution containing 15% PEG6000. Three-week-old soil-grown plants were also used for other abiotic stresses and hormone assays. For salt stress, plants were watered with 200 mM NaCl for 1 wk; for drought stress, water was withheld for 2 wk; for cold stress, the plants were transferred to 4°C for 1 d. For SA suppression of osmotic stress-induced genes, plants were sprayed with the indicated concentrations of SA before watering with PEG; for ABA treatment, plants were sprayed with 50 μM ABA.

### Microarray analysis

The detailed protocol for the microarray experiment and the raw data are available in GEO with the accession number GSE38522. Data were produced by GenePixPro 5.0 (Axon Instruments, Union City, CA, USA), imported into R 2.14 (Copenhagen Business School, Frederiksberg, Denmark) and analyzed with BioConductor (Gentleman *et al.*, 2004) using the Limma package (Smyth, 2005). Analyzed spots were background normalized using the norm-exp model from the Limma package, and then different measurement groups were quantile normalized. Our earlier analysis had shown that three-dye microarray data can have biases related to microarray fields and to different dyes. We corrected this with the modified version of ComBat (Johnson *et al.*, 2007). Fold changes were analyzed using an empirical Bayes method in Limma with an intensity-based-modified *T*-test (Sartor *et al.*, 2006). The described pipeline has a large number of free parameters and this can cause it to over-fit the model, creating a signal that is too large. We replicated the analysis with permuted sample labels. These permutations were used to perform *Z*-score normalization which compressed the signal of genes that varied a lot across the permutations. The data were next analyzed by the empirical Bayes method for significant fold change between experiments. Genes were organized into differently behaving groups and gene ontology (GO) terms. Enrichment analysis was performed using the AgriGO GO enrichment analysis tool (Du *et al.*, 2010). Gene annotations for this step were obtained from The Arabidopsis Information Resource (TAIR) website (http://www.arabidopsis.org/).

### Quantitative reverse transcription-polymerase chain reaction (qRT-PCR)

The methods used are the same as described by Besseau *et al*. (2012). The primers are listed in Supporting Information Table S2. *ACTIN2* (At3 g18780) was used as a reference gene. The qRT-PCR experiments were performed three times independently.

### Proline measurement

The proline content was determined as described by Bates *et al.* (1973) and Ramírez *et al.* (2009).

### Plant hormone (SA and ABA) measurements

Approximately 100 mg of fresh plant material were weighed, frozen in liquid nitrogen and ground with a ball mill (Retsch, Haan, Germany) in 2-ml Eppendorf tubes. The hormones were extracted twice with 10% methanol containing 1% acetic acid to which an internal standard was added (100 ng of D4-SA, 100 ng of D6-ABA), shaken for 30 min at +4°C and centrifuged for 10 min at 16 000 ***g***. The supernatants were pooled and evaporated to dryness with a concentrator (miVac, Ipswich, UK) and dissolved in 200 μl of 20% methanol. The *Arabidopsis* samples were analyzed with a Waters Acquity UPLC® system (Waters, Milford, MA, USA) equipped with a sample and binary solvent manager. In addition, a Waters Synapt GS HDMS mass spectrometer (Waters, Milford, MA, USA) was interfaced with the UPLC system via a negative electrospray ionization (ESI) source. The mass range was set from 50 to 600. Samples were analyzed in negative ion mode, with a capillary voltage of 3.0 kV. The source temperature was 120°C, the desolvation temperature was 350°C, the cone gas flow rate was 20 l h^−1^ and the desolvation gas flow rate was 1000 l h^−1^. The compounds were separated on an Acquity UPLC® BEH C18 column (Waters, Dublin, Ireland) at 40°C. The mobile phase consisted of (A) H_2_O and (B) acetonitrile (Chromasolv® grade; Sigma-Aldrich, Steinheim, Germany), both containing 0.1% HCOOH (Sigma-Aldrich). A linear gradient of eluents decreased from 95% of A to 57.4% in 4.5 min, and then increased back to 95% in 4.6 min, and was left to equilibrate for 1.4 min. The injection volume was 1 μl and the flow rate of the mobile phase was 0.6 ml min^−1^. The hormone level was determined in five independent samples for each line.

### Stomatal conductance, water loss measurements and electrolyte leakage determination

Stomatal conductance measurements were performed on both untreated and treated plant leaves with an AP4 Porometer (Delta-T Devices, Cambridge, UK). The whole-plant stomatal conductance measurements were performed as described in Kollist *et al*. (2007). For water loss measurements, leaves were detached and weighed in a plastic container at the designated time points. The percentage of water loss was calculated according to the initial measurement weight. The experiment was conducted on the laboratory bench at 55% relative humidity. Five leaves of a similar age for each line were measured.

For electrolyte leakage determination, plant materials (0.5 g) were washed with deionized water and placed in tubes with 20 ml of deionized water. The electrical conductivity of this solution (L1) was measured after 1 h of shaking at room temperature. Then, the samples were boiled for 20 min and measured a second time for conductivity (L2). The electrolyte leakage was calculated as follows: EL (%) = (L1/L2) × 100%.

### Determination of stomatal density and stomatal aperture

Epidermal peels were stripped from fully expanded leaves of 4-wk-old plants. The stomatal density was recorded under a Leitz Laborlux S microscope (Leica, Wetzlar, Germany) in 0.062 mm^2^ of leaf area. For the stomatal aperture, the stripped peels were first floated in the opening solution (containing 30 mM KCl and 10 mM MES-KOH, pH 6.15) for 2.5 h under a cool white light, and then appropriate concentrations of ABA or PEG solution were added to the opening solution. After 2 h, the stomatal apertures were measured under the microscope. The aspect ratio was determined using the image processing software ImageJ 1.43u (National Institutes of Health, Bethesda, MD, USA).

## Results

### SA-responsive *WRKY54* and *WRKY70* are induced by osmotic stress

**Figure 1:**
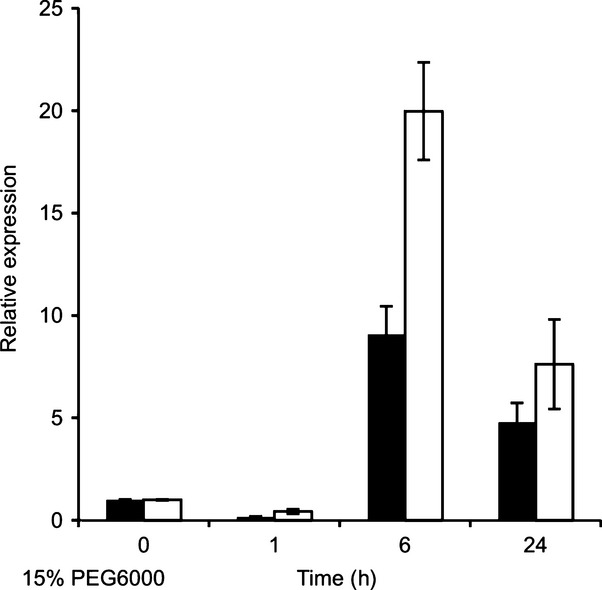
*WRKY54* (closed bars) and *WRKY70* (open bars) transcription factor genes were induced by osmotic stress. Three-week-old wild-type Arabidopsis plants grown on half-strength Murashige and Skoog (MS) solid medium were subjected to osmotic stress by transferring into half-strength MS solution with 15% polyethylene glycol (PEG)6000. Total RNA was extracted from four plants for each indicated time point (plants transferred in half-strength MS solution without PEG were used as a control) and gene expression was analyzed by quantitative reverse transcription-polymerase chain reaction (qRT-PCR). Values represent the means ± SD of three technical replicates. Three independent assays were performed with similar results.

WRKY54 and WRKY70 are key components in the establishment of plant defense (Kinkema *et al.*, 2000; Li *et al.*, 2004, 2006; Wang *et al.*, 2006). Consequently, these two TFs are rapidly induced by SA, a central mediator of plant defense against pathogens (Besseau *et al.*, 2012). To explore the possible involvement of WRKY54 and WRKY70 in abiotic stress responses, we first characterized the expression of the corresponding genes in wild-type *Arabidopsis* exposed to osmotic stress (15% PEG6000) by qRT-PCR. As shown in Fig. 1, *WRKY54* and *WRKY70* exhibited a similar early, but transient, expression pattern to osmotic stress as that induced by SA (Besseau *et al.*, 2012), with maximum induction after 6 h of PEG treatment. After 1 d, the expression of these two genes was already reduced to one-half of the maximal level.

To elucidate whether the responsiveness to osmotic stress was specific to *WRKY54* and *WRKY70*, we characterized the expression by qRT-PCR of seven additional *WRKY*s which, based on Genevestigator data (Zimmermann *et al.*, 2004), showed some response to osmotic stress. Indeed, the qRT-PCR analysis (Fig. S1) indicated that *WRKY54* and *WRKY70* are rather unique among the *WRKY*s tested in their rapid and prominent induction by osmotic stress. The other two *WRKY*s clearly induced by osmotic stress were *WRKY63* and *WRKY40*, which have been implicated previously in osmotic stress adaptation (Ren *et al.*, 2010; Shang *et al.*, 2010). However, these genes showed a different temporal pattern of expression with delayed and more persistent induction relative to *WRKY54* and *WRKY70*.

### Inactivation of *WRKY54* and *WRKY70* enhances plant tolerance to osmotic stress

**Figure 2:**
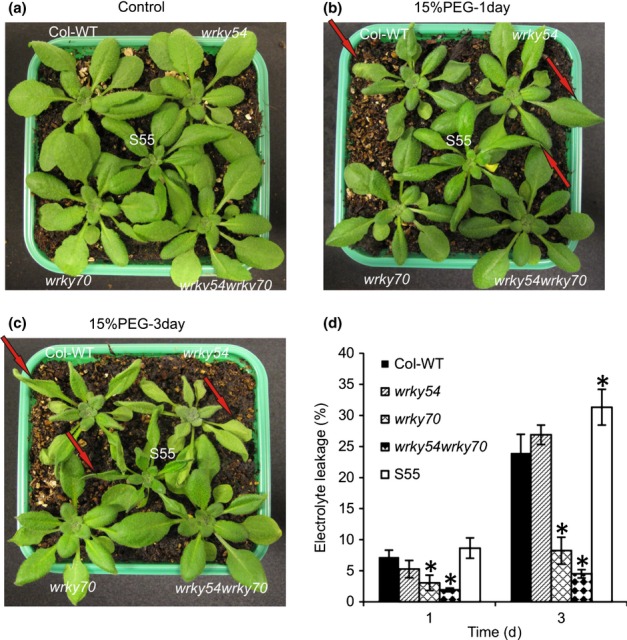
Osmotic stress tolerance of mutants and transgenic Arabidopsis affected in *WRKY54* and *WRKY70* expression. (a–c) Three-week-old Arabidopsis plants were subjected to osmotic stress by watering with 15% polyethylene glycol (PEG)6000 for 3 d. Wild-type plants (Col-WT), *wrky54* and *wrky70* single mutants, the *wrky54wrky70* double mutant and the *WRKY70* overexpressor line (S55) were grown together to compare plants with equivalent osmotic stress treatment. Eight replicates of the combination were tested with similar results. Only one representative pot was used to take photographs on the first and third day after treatment. Red arrows indicate the wilting symptoms at the tips and edges of the leaves on the first day, with subsequent spread to the whole leaves on the third day. This assay was repeated three times with similar results. (d) Electrolyte leakage assay was performed on leaves after exposure to 15% PEG for 1 and 3 d. Five replicates of each line were used for conductivity measurement. Error bars indicate ± SD (*, *P *<* *0.01, one-way ANOVA test).

To explore the possible involvement of WRKY54 and WRKY70 in osmotic stress tolerance, wild-type plants (Col-WT), *wrky54* and *wrky70* single and double mutants, as well as a *WRKY70* overexpressor line (S55), were exposed to osmotic stress by watering the plants with 15% PEG6000. Plant phenotypes were observed 1 and 3 d later (Fig. 2a–c). Following PEG treatment, the *wrky54wrky70* double-mutant plants showed markedly enhanced tolerance to osmotic stress, whereas wild-type plants showed classic symptoms of wilting, especially at the leaf margins on the first day. Subsequently, the wilted symptoms in the wild-type spread to the whole leaves after 3 d, whereas the *wrky54wrky70* double mutant still exhibited enhanced tolerance (Fig. 2b,c). In comparison, after 3 d, the *wrky54* single mutant showed equivalent symptoms to wild-type plants, whereas the *wrky70* single mutant presented a less wilted phenotype than the wild-type, but not the tolerance exhibited by the *wrky54wrky70* double mutant. By contrast, the transgenic line overexpressing *WRKY70* became clearly wilted on osmotic stress, especially on the third day (Fig. 2b,c).

To quantify the stress damage, ion leakage was measured during stress exposure (Fig. 2d). Electrolyte leakage was increased rapidly in the wild-type and *wrky54* single mutant during exposure to stress, whereas the *wrky54wrky70* double mutant showed very low electrolyte leakage, in accordance with the observed visual plant phenotypes. The *wrky70* single mutant presented an intermediate loss of ions, whereas the *WRKY70* overexpressor exhibited the opposite phenotype, with a considerably higher electrolyte leakage than the other lines (Fig. 2d).

These results demonstrate that inactivation of both *WRKY54* and *WRKY70* enhances plant tolerance to osmotic stress, and suggest that these two TFs co-operate as negative regulators of osmotic stress tolerance.

### Osmotic stress-induced expression of abiotic stress response genes is suppressed in *wrky70* and *wrky54* mutants

To explore the possible causes of the enhanced tolerance to osmotic stress observed in the *wrky54wrky70* double mutant, we characterized global gene expression by microarray experiments using an Agilent *Arabidopsis* (V4) Gene Expression Microarray (Palo Alto, CA, USA), which contains 43 803 probe sets. Global gene expression patterns in unstressed wild-type plants were compared with those from wild-type and *wrky54wrky70* mutant plants exposed to osmotic stress (15% PEG6000). Among the 43 803 probe sets, over 900 probes showed marked induction (log_2_FC ≥ 1.5) by osmotic stress in wild-type plants. GO enrichment analysis highlighted 70 significant GO terms classified as biological process (P), molecular function (F) or cellular component (C) (Table S1). As assumed, the majority of the GO terms could be assigned to response to stimulus and abiotic stress. The abiotic stimulus GO class 0009628 contained 97 genes, from which 58 representative genes were used for the comparison between mutant and wild-type plants under osmotic stress (Table 1). These 58 genes contained ABA-responsive genes and genes for heat shock proteins, oxidative stress-related proteins and several TFs. Interestingly, PEG induction of these genes was drastically reduced or suppressed in the *wrky54wrky70* double mutant relative to that observed in wild-type plants (Table 1). Inactivation of *WRKY54* and *WRKY70* genes thus appears to block the induction of abiotic stress-related genes by osmotic stress.

**Table 1:** Comparison of osmotic stress-related gene expression in Arabidopsis wild-type plants (Col-WT) and the *wrky54wrky70* double mutant under 15% polyethylene glycol (PEG) treatment for 1 d; the expression level in Col-WT without any treatment was used as a control

AGILENT_ID	Description	AGI number	Col-WT-1 d vs Col-WT-ctrl		*wrky54wrky70*-1 d vs Col-WT-ctrl	
			log_2_FC	*P* value	log_2_FC	*P* value
A_84_P255380	Unknown protein	AT1G16850	5.61	6.85E-06	2.09	2.57E-03
A_84_P13852	Heat shock protein 21 (HSP21)	AT4G27670	5.09	4.43E-07	2.01	1.99E-03
A_84_P19363	Heat shock protein 17,4 (ATHSP17,4)	AT3G46230	5.07	3.91E-06	2.65	2.93E-04
A_84_P10874	Low temperature-induced 30 (LTI30)	AT3G50970	5.01	2.76E-04	2.08	7.38E-03
A_84_P21625	Responsive to desiccation 29B (LTI65/RD29B)	AT5G52300	4.96	1.38E-05	2.37	4.32E-03
A_84_P19758	Lipid transfer protein 4 (LTP4)	AT5G59310	4.67	3.96E-06	2.87	1.05E-04
A_84_P21525	Heat shock protein 17,6A (HSP17,6A)	AT5G12030	4.55	4.06E-07	2.33	1.75E-04
A_84_P18335	Abscisic acid (ABA)-responsive protein	AT3G02480	4.25	3.14E-04	2.13	1.48E-03
A_84_P20579	Heat shock protein (HSP17,6II)	AT5G12020	4.24	1.72E-08	1.89	1.89E-06
A_84_P11248	Protein phosphatase 2C (PP2C)	AT5G59220	4.09	5.96E-08	2.86	1.74E-06
A_84_P14587	Nine-cis-epoxycarotenoid dioxygenase3 (NCED3)	AT3G14440	4.09	4.63E-07	2.44	3.49E-06
A_84_P597426	Heat-stress-associated 32 (HSA32)	AT4G21320	3.80	2.34E-07	0.83	3.46E-03
A_84_P23658	Alcohol dehydrogenase 1 (ADH1)	AT1G77120	3.65	1.29E-06	1.44	2.33E-04
A_84_P12209	ABA and stress-inducible protein (ATHVA22B)	AT5G62490	3.57	2.29E-06	2.18	1.53E-05
A_84_P811915	Responsive to ABA 18 (RAB18)	AT5G66400	3.57	4.86E-07	1.09	5.12E-02
A_84_P12012	SNF1-related protein kinase 2,7 (SNRK2-7)	AT4G40010	3.50	1.03E-07	2.22	2.49E-06
A_84_P17859	Cell wall-modifying enzyme/hydrolase protein 22 (TCH4)	AT5G57560	3.33	7.37E-06	1.78	3.99E-04
A_84_P19166	UDP-glycosyltransferase (UGT73C1)	At2G36750	3.24	1.56E-07	1.57	8.41E-06
A_84_P14854	Drought-induced protein (ATDI21)	AT4G15910	3.13	5.96E-07	0.82	3.20E-03
A_84_P23852	Cold-regulated 15A (COR15A)	AT2G42540	3.09	1.96E-05	0.75	1.15E-02
A_84_P162633	S2P-like putative metalloprotease (ATEGY3)	AT1G17870	3.01	7.03E-07	0.62	1.06E-02
A_84_P12765	Dehydrin xero1 (XERO1)	AT3G50980	2.94	2.54E-05	0.73	3.50E-02
A_84_P11587	Delta1-pyrroline-5-carboxylate synthase 1 (P5CS1)	AT2G39800	2.92	6.00E-06	1.76	3.03E-04
A_84_P10384	Cold regulated 47 (COR47)	AT1G20440	2.91	2.08E-05	1.04	5.89E-03
A_84_P22571	Low temperature-induced 78 (LTI78)	AT5G52310	2.81	8.86E-06	0.91	1.00E-02
A_84_P10659	Homeobox protein 12 (ATHB-7)	AT2G46680	2.79	1.58E-06	1.79	5.36E-05
A_84_P18401	Heat shock protein 70	AT3G12580	2.79	1.23E-06	0.64	4.82E-03
A_84_P18845	MYB family transcription factor (MYB112)	At1G48000	2.72	4.29E-07	1.84	1.91E-05
A_84_P22020	Beta-ketoacyl-CoA synthase family protein (KCS3)	AT1G07720	2.65	3.74E-06	1.13	3.10E-04
A_84_P10151	Beta-ketoacyl-CoA synthase family protein (KCS19)	AT5G04530	2.65	5.00E-07	1.36	6.56E-05
A_84_P11961	Responsive to desiccation 26 (RD26)	AT4G27410	2.57	8.91E-07	1.44	8.15E-05
A_84_P23992	Protein phosphatase 2C (PP2C)	AT3G05640	2.47	6.26E-07	1.60	4.06E-05
A_84_P11342	Late embryogenesis abundant 14 (LEA14)	AT1G01470	2.41	5.19E-05	1.03	8.06E-05
A_84_P14827	Arginine decarboxylase 2 (ADC2)	AT4G34710	2.40	1.71E-06	1.75	1.03E-05
A_84_P10469	CCAAT-binding transcription factor (CBF-B/NF-YA)	AT1G54160	2.36	5.25E-07	0.45	1.18E-02
A_84_P21874	Salt tolerance finger protein (STZ)	AT1G27730	2.35	1.45E-05	1.84	3.84E-05
A_84_P117182	Rare-cold-inducible 2B protein (RCI2B)	AT3G05890	2.33	2.41E-05	0.49	2.43E-02
A_84_P15486	Rare-cold-inducible 2A protein (RCI2A)	AT3G05880	2.32	1.10E-06	0.40	1.50E-02
A_84_P62840	Early light-inducible protein 2 (ELIP2)	AT4G14690	2.31	1.56E-06	1.37	4.98E-05
A_84_P11731	DNA binding/transcription coactivator (ATMBF1C/MBF1C)	AT3G24500	2.27	1.12E-05	0.80	5.95E-04
A_84_P10555	Heat shock protein (HSP17,6C-CI)	AT1G53540	2.26	2.00E-05	1.71	8.93E-05
A_84_P18803	ABA insensitive 2 (ABI2)	AT5G57050	2.22	1.19E-06	1.10	1.14E-03
A_84_P810688	Cold and ABA-inducible protein KIN1	AT5G15960	2.20	1.05E-04	0.52	1.35E-02
A_84_P10949	MYB domain protein 74 (AtMYB74)	AT4G05100	2.14	1.98E-06	1.81	1.07E-05
A_84_P22572	Heat shock protein (HSP81-1)	AT5G52640	2.05	4.77E-06	1.62	5.93E-05
A_84_P275730	ABI five binding protein 4 (TMAC2/AFP4)	AT3G02140	2.01	2.67E-06	1.81	1.91E-05
A_84_P15646	Homeobox protein 12, transcription factor (ATHB-12)	AT3G61890	1.94	1.47E-06	0.82	3.33E-04
A_84_P53000	Responsive to desiccation 2 (RD2)	AT2G21620	1.87	7.37E-06	0.83	1.34E-03
A_84_P16040	Early-responsive to dehydration 7 (ERD7)	AT2G17840	1.84	9.08E-06	0.59	2.53E-03
A_84_P18269	Dehydrin lea (LEA)	AT2G21490	1.79	5.30E-04	0.74	1.97E-03
A_84_P11439	Heat shock protein-like (HSP26,5-P)	AT1G52560	1.79	3.01E-06	0.35	2.46E-02
A_84_P24127	Universal stress family protein	AT3G53990	1.75	3.19E-06	0.91	2.12E-04
A_84_P10318	Myb domain protein 96 (MYB96)	AT5G62470	1.72	6.10E-06	1.01	4.00E-04
A_84_P166453	Interferon-related developmental regulator family protein	AT1G27760	1.68	2.77E-06	0.59	1.46E-03
A_84_P13675	Calcium-dependent, membrane-binding protein (ANNAT1)	AT1G35720	1.65	1.83E-05	0.48	7.58E-03
A_84_P18573	ABA insensitive 1 (ABI1)	AT4G26080	1.64	4.52E-06	1.12	5.87E-05
A_84_P13757	Phytochrome interacting factor3-like 2 protein (PIL2)	AT3G62090	1.64	6.96E-04	0.98	1.44E-03
A_84_P17787	Heat shock protein-like (HSP15,7-CI)	AT5G37670	1.64	6.57E-05	0.55	6.37E-03
A_84_P714600	Zinc-finger protein 2 (AZF2)	AT3G19580	1.63	2.68E-03	1.38	3.55E-03

**Figure 3:**
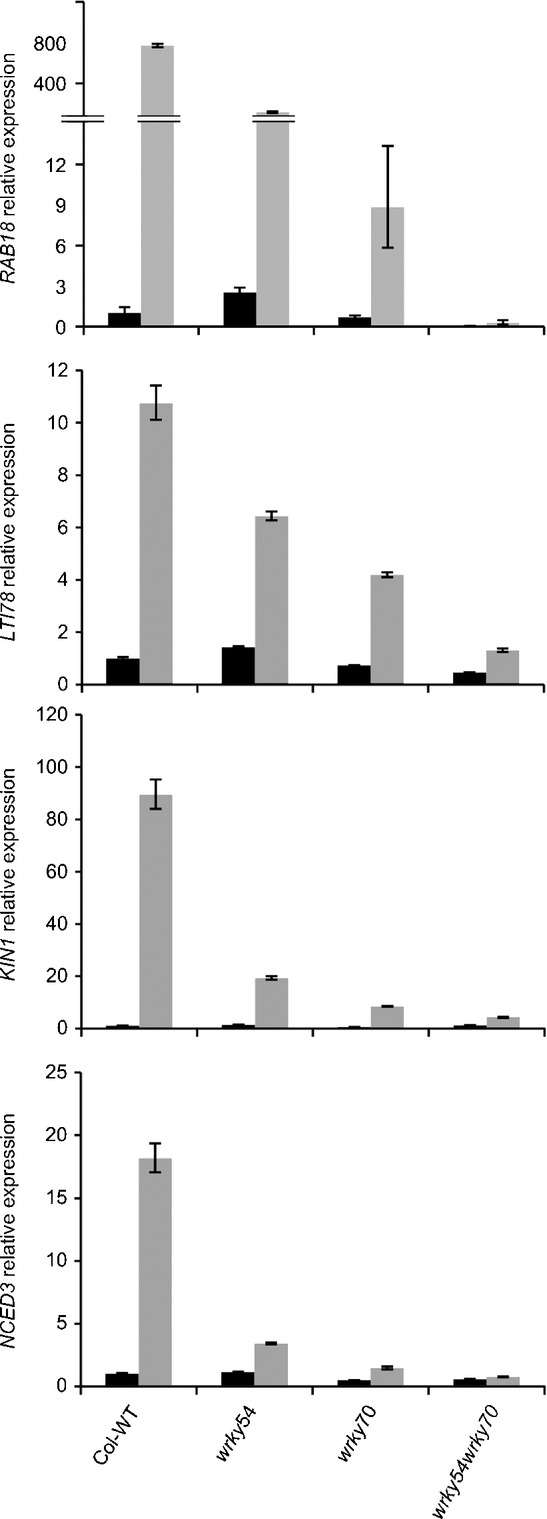
Expression of osmotic stress-responsive genes in Arabidopsis wild-type (Col-WT) and *wrky54*, *wrky70* and *wrky54wrky70* mutants, assayed by quantitative reverse transcription-polymerase chain reaction (qRT-PCR). Three-week-old plants were stressed by watering with 15% polyethylene glycol (PEG)6000. Leaves from untreated (control; black bars) and treated (grey bars) plants were collected at the 1 d time point. The relative expression of each gene was normalized to that of *ACT2*. Values were obtained from the means ± SD of three technical replicates. Three independent assays were performed with similar results.

To verify these intriguing results from microarray experiments, the expression of typical abiotic stress-inducible marker genes was characterized in mutants and wild-type plants by qRT-PCR (Fig. 3). The tested genes included *RAB18*, *LTI78* and *KIN1* induced by ABA, drought and low temperature (Kurkela & Franck, 1990; Lång & Palva, 1992; Nordin *et al.*, 1993), as well as *NCED3*, encoding a key enzyme in ABA biosynthesis (Iuchi *et al.*, 2001). As shown in Fig. 3, all the tested genes were highly induced in wild-type plants in response to osmotic stress (by watering with 15% PEG6000 for 1 d), whereas the induced expression level was dramatically reduced in both the single mutants and, especially, in the *wrky54wrky70* double mutant. The reduced expression of these osmotically induced genes in *wrky* mutants indicates a requirement for *WRKY54* and *WRKY70* in the induction of osmotic stress-responsive genes, which is in contradiction with the osmotic stress tolerance observed in the *wrky54wrky70* mutants.

### Proline content is reduced in the *wrky54wrky70* double mutant

**Figure 4:**
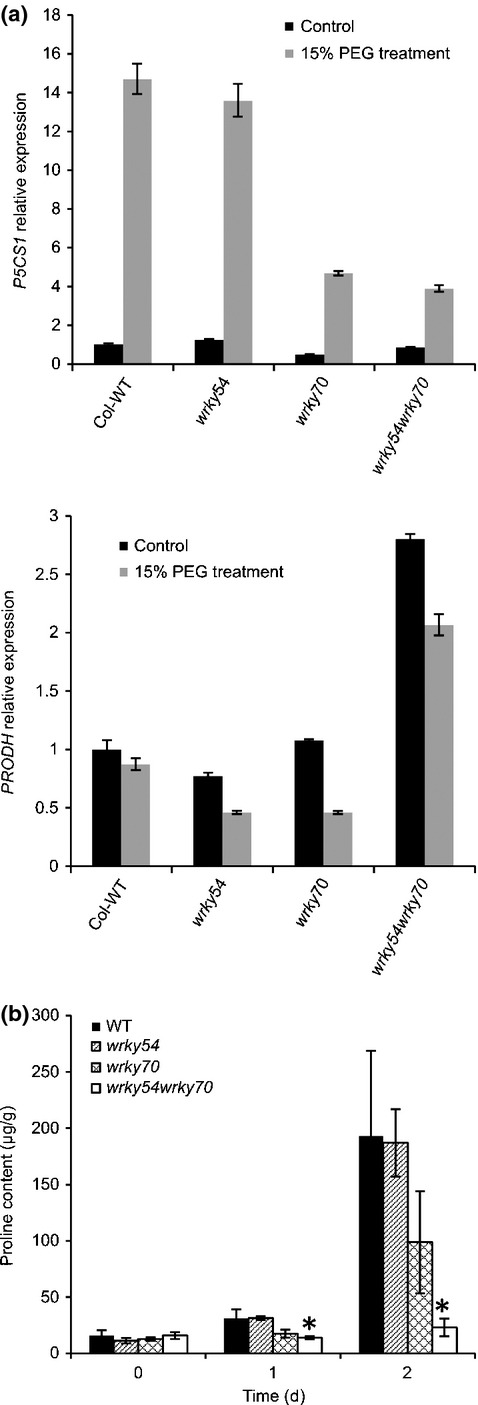
Proline metabolism under osmotic stress in Arabidopsis wild-type (Col-WT) and *wrky54*, *wrky70* and *wrky54wrky70* mutants. (a) The expression of proline-related genes *P5CS1* and *ProDH* was analyzed by quantitative reverse transcription-polymerase chain reaction (qRT-PCR). Three-week-old plants were treated by watering with 15% polyethylene glycol (PEG)6000 for 1 d (gray bars). Untreated plants were used as the control (black bars). The relative gene expression was calculated and normalized with the reference gene *ACT2*. Error bars represent the standard deviations from three technical replicates and three independent assays were performed with similar results. (b) Free proline contents were measured on 3-wk-old plants under osmotic stress (watered with 15% PEG6000) after 1 and 2 d. Four replicates of each line were used to calculate the standard deviation. Three independent assays were performed with similar results. Error bars indicate ± SD (*, *P *<* *0.01, one-way ANOVA test).

Osmotic stress tolerance is associated with the accumulation of osmoprotectants, such as proline (Delauney & Verma, 1993). To explore whether the increased tolerance to osmotic stress in the *wrky54wrky70* double mutant could be dependent on proline accumulation, we characterized the expression of genes in proline metabolism as well as proline content. We first monitored the expression of the proline-related genes *P5CS1* and *ProDH* by qRT-PCR (Fig. 4a). *P5CS1* and *ProDH* encode the rate-limiting enzymes for proline biosynthesis and catabolism, respectively (Nakashima *et al.*, 1998; Yoshiba *et al.*, 1999). As shown by the qRT-PCR results (Fig. 4a), the induction of *P5CS1* was strongly reduced in *wrky70* and *wrky54wrky70* mutants under osmotic stress relative to the wild-type and *wrky54* single mutant. By contrast, the expression of *ProDH*, required for proline degradation, was already up-regulated in the *wrky54wrky70* mutant without stress. Consistent with the gene expression data (Fig. 4a), measurement of the proline content showed that osmotically induced proline accumulation was abolished in the *wrky54wrky70* double mutant (Fig. 4b). Once again, an intermediate effect was observed in the *wrky70* mutant and no significant difference was found for the *wrky54* mutant relative to the wild-type. These results show that inactivation of *WRKY54* and *WRKY70* genes leads to reduced expression of proline biosynthesis and enhanced expression of proline degradation genes and, consequently, impaired accumulation of proline under osmotic stress. Taking the microarray and proline data together, the osmotic stress tolerance exhibited by the *wrky54wrky70* double mutant could not be explained by either the expression of stress-related genes or the accumulation of the osmoprotectant proline.

### Involvement of SA in *wrky54wrky70*-dependent osmotic stress tolerance

**Figure 5:**
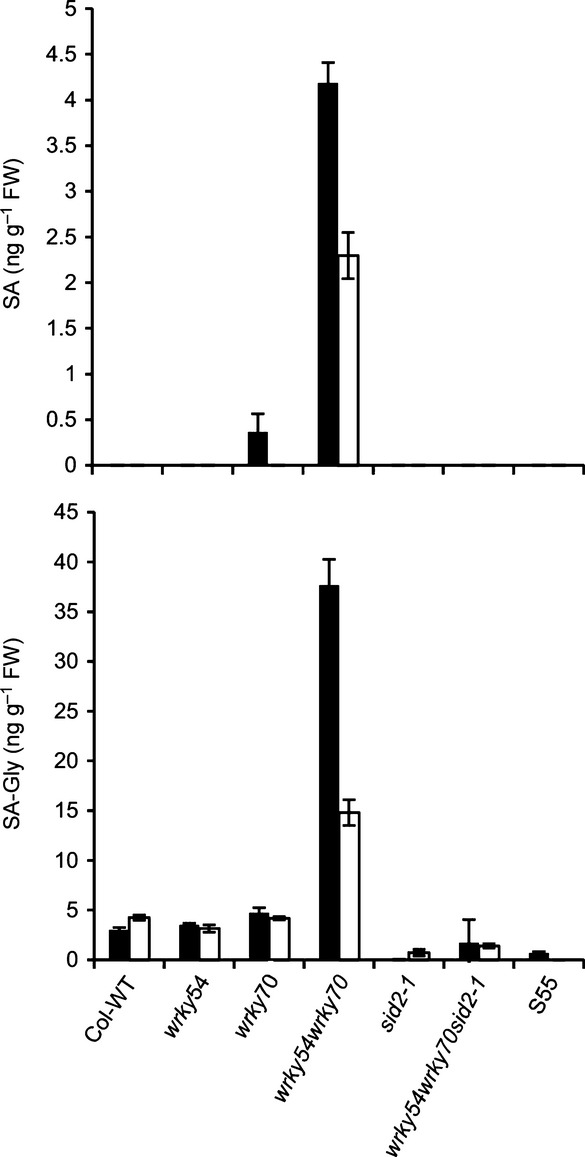
Salicylic acid (SA) levels in Arabidopsis wild-type (Col-WT), *wrky54*, *wrky70*, *wrky54wrky70*, *sid2-1* and *wrky54wrky70sid2-1* mutants, and the *WRKY70*-overexpressing line (S55) under osmotic stress. The levels of free SA and SA glucosides (SAG) in the leaves of 4-wk-old soil-grown plants were analyzed by UPLC. Analysis was performed on untreated plants (control, closed bars) and after 15% polyethylene glycol (PEG)6000 watering for 1 d (open bars). Values are mean ± SD of five individual replicates of each line.

WRKY54 and WRKY70 are well known to be involved in plant defense signaling, positively regulated by SA through the receptor NPR1 and its paralogs NPR3 and NPR4 (Fu *et al.*, 2012; Wu *et al.*, 2012). Consequently, *wrky54wrky70* double mutants are impaired in plant defense against phytopathogens (Li *et al.*, 2004, 2006; Wang *et al.*, 2006). In addition, the double mutants present an enhanced level of free SA, indicating a dual function for both WRKY54 and WRKY70 as negative regulators of SA biosynthesis (negative feedback), in addition to the regulation of SA-mediated gene expression (Wang *et al.*, 2006). To explore the possible correlation between the osmotic stress tolerance and alteration in endogenous hormone synthesis, we measured both free SA and SA glucoside (SAG) levels in different genotypes under osmotic stress (Fig. 5). We included in the analysis the *sid2-1* mutant defective in isochorismate synthase and consequently impaired in SA biosynthesis (Wildermuth *et al.*, 2001). The basal levels of both free SA and SAG were clearly elevated in the *wrky54wrky70* double mutant relative to the other lines (Fig. 5), consistent with previous results. Interestingly, this enhanced accumulation was abolished by the introduction of the *sid2-1* allele into the *wrky54wrky70* background. Indeed, the triple mutant *wrky54wrky70sid2-1* exhibited free SA and conjugated SA levels similar or even lower than those of the wild-type (Fig. 5). Finally, exposure to osmotic stress reduced the high SA levels in the *wrky54wrky70* double mutant relative to those in the non-stressed control (Fig. 5).

**Figure 6:**
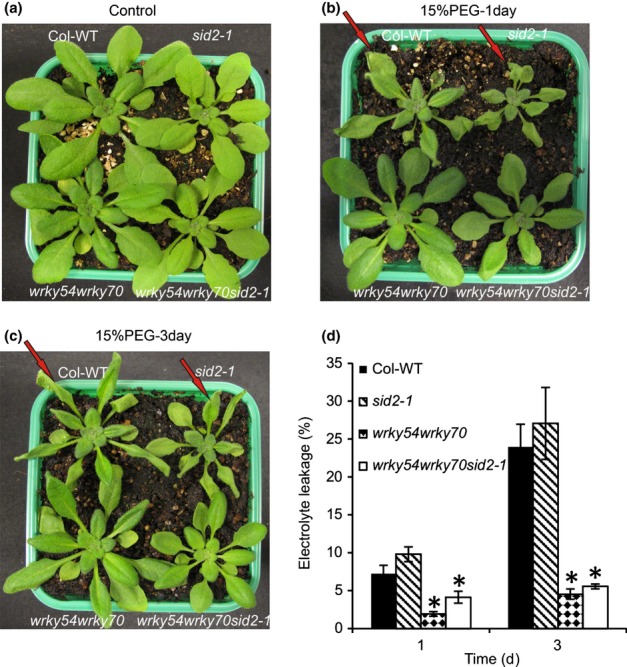
Osmotic stress tolerance in *wrky54wrky70* was not suppressed by the introduction of *sid2-1*. (a–c) Three-week-old plants of Arabidopsis wild-type (Col-WT) and *sid2-1*, *wrky54wrky70* and *wrky54wrky70sid2-1* mutants were exposed to osmotic stress treatment by watering with 15% polyethylene glycol (PEG)6000 for 3 d. Each genotype was grown in the same pot to perform equivalent treatments. Eight pots were used at the same time and the photographs were taken 1 and 3 d after treatment. Red arrows indicate the wilting symptoms at the tips and edges of the leaves on the first day, with subsequent spread to the whole leaves on the third day. (d) Electrolyte leakage was assessed on leaves after exposure to 15% PEG for 1 and 3 d. Five replicates of each line were used for conductivity measurement. Error bars indicate ± SD from five replicates (*, *P *<* *0.01, one-way ANOVA test).

The reduction of high SA levels by the introduction of *sid2-1* into *wrky54wrky70* did not abolish the enhanced tolerance of these mutants, although a slight reduction in tolerance was visible in the triple mutant when compared with the double mutant (Fig. 6a–c). The electrolyte leakage in the *wrky54wrky70sid2-1* triple mutant under osmotic stress was clearly reduced when compared with that of the wild-type and almost reached that of *wrky54wrky70* after 3 d (Fig. 6d). These results suggest that SA over-accumulation is not responsible for the enhanced osmotic stress tolerance observed in *wrky54wrky70* lines. However, SA accumulation in the *wrky54wrky70* mutant could explain the suppression in the expression of the osmotic stress response genes observed. Indeed, the introduction of *sid2-1* into the *wrky54wrky70* mutant background restored the induction of stress-responsive genes close to wild-type levels (Table S3). In order to support our presumption that SA suppresses the expression of osmotic stress-induced genes, we tested the effect of exogenous SA on PEG-induced expression of *RAB18*, *LTI78*, *KIN1* and *NCED3* in wild-type plants (Fig. S2). Osmotically induced expression of these genes was clearly decreased by exogenous SA in a concentration-dependent manner. These results support the hypothesis that suppression of the expression of osmotic stress-related genes in the *wrky54wrky70* double mutant is indeed a consequence of the enhanced SA levels in this mutant.

### Inactivation of *WRKY54* and *WRKY70* enhances plant tolerance to abiotic stresses

Osmotic stress can be caused by several environmental cues, such as drought, high salinity and low temperature. To elucidate whether the tolerance caused by the inactivation of *WRKY54* and *WRKY70* was specific to PEG-treated plants, or could also result from other abiotic factors, the wild-type and mutant plants were exposed to high-salt, drought and low-temperature stresses. We first explored the response of the two *WRKY* genes to these cues as well as to exogenous ABA (Fig. S3). Our results of this gene expression analysis by qRT-PCR showed a transient induction of both genes, similar to that seen in response to PEG (Fig. 1). To determine the role of these WRKY TFs in Arabidopsis stress tolerance, we characterized the phenotypes and stress damage by ion leakage from plants exposed to these stress conditions (Figs S4, S5). In accordance with data from PEG-treated plants (Fig. 2), we observed clearly enhanced tolerance to drought stress in both double and triple mutants and also somewhat improved tolerance to high salinity. However, we did not observe any significant increase in freezing tolerance of the plants (data not shown). Taken together, these data suggest that the results obtained with PEG-treated plants also apply to natural abiotic stresses, such as drought stress.

### Osmotic stress tolerance of *wrky54wrky70* mutants is caused by enhanced stomatal closure

**Figure 7:**
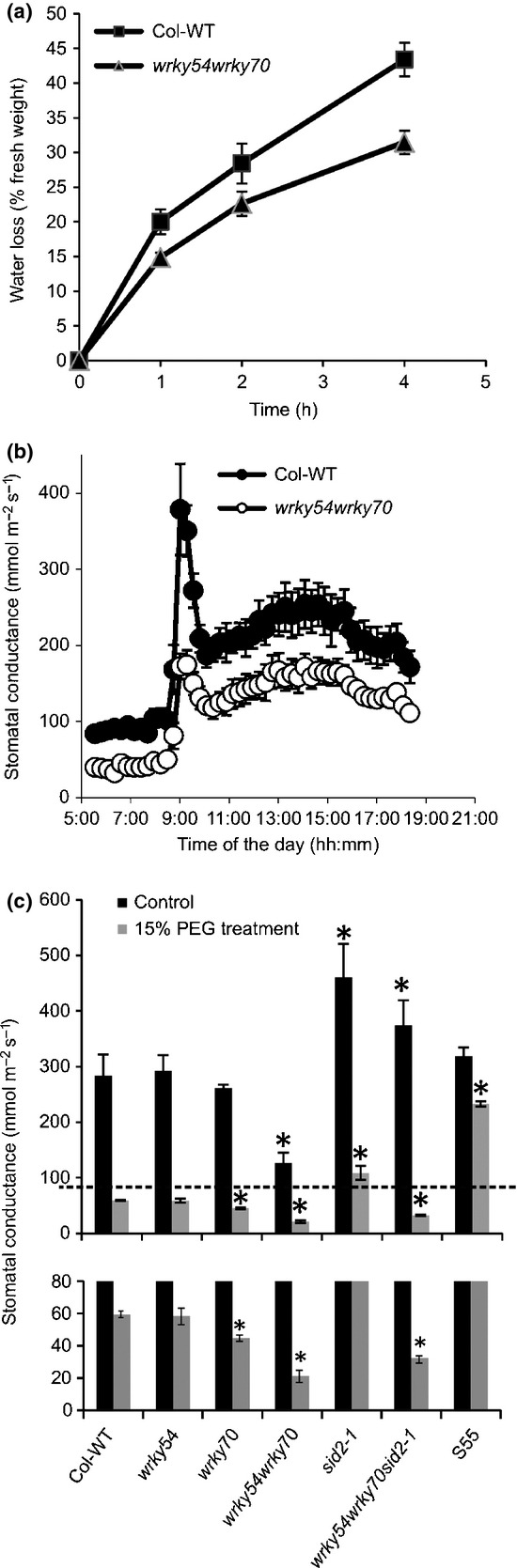
Enhanced osmotic stress tolerance in *wrky54wrky70* could be explained by the lower water loss rate and more efficient stomatal closure. (a) Determination of the water loss of excised leaves from 4-wk-old Arabidopsis. Values are mean ± SD; five different leaves per line at a similar stage were used for the experiments. Three independent experiments were performed. (b) Whole-plant stomatal conductance was measured in 4-wk-old and non-treated Arabidopsis (*n *= 4, ± SEM). (c) Stomatal conductance in 4-wk-old Arabidopsis was measured before (black bars) and after 1 d of 15% polyethylene glycol (PEG) treatment (gray bars). Three leaves of similar size picked from all eight plants were measured for each line. Values are mean ± SD (*, *P *<* *0.01, one-way ANOVA test). The results were based on three independent experiments. S55 represents the *WRKY70* overexpressor line. The bottom figure represents the lower part of the graph (below the dashed line) with the *y*-axis expanded to better visualize the differences in conductance.

In response to drought or osmotic stress, plants are able to control their water content and reduce water loss. As genes responsive to osmotic stress and osmoprotectants were not implicated in osmotic stress tolerance of the *wrky54wrky70* mutants, we explored the involvement of water balance regulation to explain the observed tolerance phenotype. To monitor plant water loss, we measured the weight loss of excised leaves (Fig. 7a). Leaves of the *wrky54wrky70* double mutant exhibited significantly lower water loss than those of wild-type plants, highlighting the improved capacity of the mutant to retain water. As water loss is mainly controlled by stomata (Verslues *et al.*, 2006), we subsequently explored stomatal regulation as a possible explanation for the observed stress tolerance phenotypes. To achieve this, we first compared the number of stomata per unit leaf area between wild-type and *wrky54wrky70* plants, but no significant differences were detected (Fig. S6). Then, we measured stomatal conductance in untreated (Fig. 7b) and osmotically stressed (Fig. 7c) plants to explore possible alterations in stomatal movement. Interestingly, the *wrky54wrky70* double mutant exhibited drastically reduced stomatal conductance in both control and PEG-treated plants relative to the other lines. This indicates that the double mutant has more closed stomata relative to the wild-type plants. Moreover, exposure to osmotic stress resulted in further enhanced stomatal closure in the double mutant relative to that of wild-type plants. Consequently, the reduced stomatal conductance in the *wrky54wrky70* mutant could explain the observed osmotic stress tolerance. Interestingly, in contrast with the *wrky54wrky70* mutant, the corresponding single mutant *wrky70* exhibited only slightly lower stomatal conductance, whereas *wrky54* did not show any significant difference relative to the wild-type, indicating co-operation between WRKY54 and WRKY70 in the control of stomatal conductance. Accordingly, the *WRKY70* overexpression line (S55) displayed somewhat higher stomatal conductance than the wild-type under non-stressed conditions and, in contrast with the wild-type, was clearly impaired in stomatal closure in response to osmotic stress. Taken together, these data suggest that WRKY54 and WRKY70 co-operate as negative regulators of stomatal closure.

Our data (Fig. 6) indicated that the elevated SA levels in the *wrky54wrky70* double mutant contributed only weakly to the osmotic stress tolerance phenotype, but, as SA has recently been implicated in the control of stomatal movement (Khokon *et al.*, 2011), we explored the possible contribution of SA to the enhanced stomatal closure in the *wrky54wrky70* mutants. Both the *wrky54wrky70sid2-1* triple mutant and the *sid2-1* single mutant used as a control showed enhanced stomatal conductance under non-stressed conditions (Fig. 7c), possibly caused by the reduced SA levels in the *sid2-1* background (Fig. 5). However, the lack of SA in the triple mutant as a result of the *sid2-1* mutation did not have any major effect on the enhanced stomatal closure observed in the *wrky54wrky70* background exposed to osmotic stress. This was in contrast with the *sid2-1* single mutant, which exhibited reduced stomatal closure under osmotic stress relative to the wild-type.

**Figure 8:**
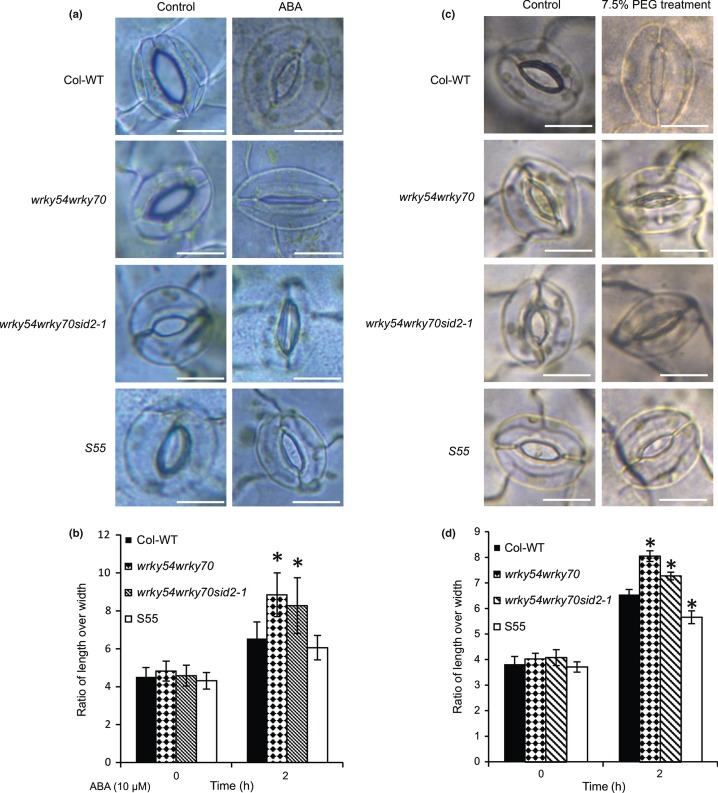
Fast stomatal response to abscisic acid (ABA) and polyethylene glycol (PEG). (a) Comparison of stomatal aperture in response to ABA. Scale bar = 10 μm. (b) Ratio of stomatal aperture length over width. Data were calculated from 100 stomata from leaves of three different plants of Arabidopsis. Values are mean ± SD. The experiments were performed three times with similar results (*, *P *<* *0.01, one-way ANOVA test). (c) Comparison of stomatal aperture in response to PEG treatment. Scale bar = 10 μm. (d) Ratio of stomatal aperture length over width. Data were calculated from 100 stomata of leaves of three different plants of Arabidopsis. Values are mean ± SD. The experiments were performed three times with similar results (*, *P *<* *0.01, one-way ANOVA test).

In addition to the adaptive stomatal responses (triggered by 1 d of PEG exposure) presented above (Fig. 7c), fast responses may also be affected in the *wrky54wrky70* double-mutant background. To elucidate the effect of *WRKY54* and *WRKY70* on fast stomatal responses, we measured the stomatal apertures in response to both PEG and exogenous ABA. The results (Fig. 8) suggest that the *WRKY* genes are also involved in fast stomatal responses triggered either by osmotic stress or ABA and, in accordance with the results from adaptive studies, suggest that the inactivation of both *WRKY* genes promotes stomatal closure, whereas overexpression of *WRKY70* seems to have an opposite effect.

**Figure 9:**
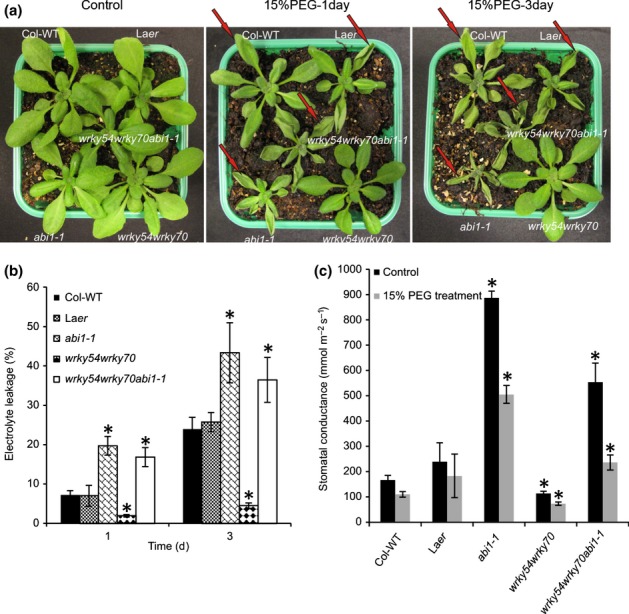
Osmotic stress tolerance in *wrky54wrky70abi1-1* triple mutant was abolished. (a) Three-week-old plants of Arabidopsis were exposed to osmotic stress treatment using 15% polyethylene glycol (PEG)6000 for 3 d. Plants treated with water were used as a control. Wild-type plants (Col-WT and Ler-WT), *abi1-1* single mutant, *wrky54wrky70* double mutants and *wrky54wrky70abi1-1* triple mutant were grown together in one pot. Eight replicates for this combination were performed with similar results. Only one representative pot was used to take photographs on the first and third days after treatment. Red arrows indicate the wilting symptoms at the tips and edges of the leaves on the first day, with subsequent spread to the whole leaves on the third day. This assay was repeated three times. (b) Electrolyte leakage was assessed on leaves after exposure to 15% PEG for 1 and 3 d. Five replicates of each line were used for conductivity measurement. Error bars indicate ± SD (*, *P *<* *0.01, one-way ANOVA test). (c) Stomatal conductance of 4-wk-old Arabidopsis was measured before (black bars) and after (gray bars) 1 d of 15% PEG treatment. Three leaves of similar size picked from all eight plants were measured for each line. Values are mean ± SD. The results were based on three independent experiments (*, *P *<* *0.01, one-way ANOVA test).

The central phytohormone that controls the stomatal aperture is ABA (Raghavendra *et al.*, 2010). To explore the role of ABA in the WRKY-mediated stomatal control, we introduced the dominant negative *abi1-1* mutation into the *wrky54wrky70* double mutant. ABI1 is a key component in the ABA signal transduction pathway (Moes *et al.*, 2008). In accordance with the importance of ABA signaling in the osmotic stress response, the *abi1-1* single mutant was much more strongly affected than the corresponding wild-type by exposure to PEG (Fig. 9a), with increased ion leakage and higher stomatal conductance (Fig. 9b,c). Remarkably, the osmotic tolerance observed in the *wrky54wrky70* double mutant was clearly reduced by the introduction of the *abi1-1* allele (Fig. 9a). Similarly, the observed reduction in electrolyte leakage under osmotic stress in the *wrky54wrky70* background was abolished (Fig. 9b). Similar results were obtained for stomatal conductance, with a clear increase in conductance in the *wrky54wrky70abi1-1* mutant when compared with that of the double mutant, under both unstressed and osmotically stressed conditions (Fig. 9c), highlighting the central role and requirement for intact ABA signaling in stomatal control (Hetherington, 2001). To further explore the role of ABA in the altered stomatal control of the *wrky* mutants and the *WRKY70* overexpressor, we characterized the level of ABA in these backgrounds by air drying leaves for 2 h (Fig. S7). The results did not show a statistically significant increase in ABA in the *wrky54wrky70* double mutant in unstressed or stressed plants. However, the ABA level was clearly decreased in the overexpressor line, suggesting a possible explanation for the impaired ability of this line to close its stomates.

## Discussion

### WRKY70 and WRKY54 co-operate as negative regulators of the osmotic stress response

WRKY70 and its closest homolog WRKY54 have been best characterized for their function in the regulation of systemic acquired resistance and innate immunity in plants. They behave as positive regulators of SA-mediated gene expression and as negative regulators of SA biosynthesis (Li *et al.*, 2004, 2006; Wang *et al.*, 2006). Recently, we have shown that, additionally, these two TFs co-operate as negative regulators in developmental senescence (Besseau *et al.*, 2012). Prompted by the induction of these genes by abiotic stress (Fig. 1), and to expand our previous analysis of the biological roles of these TFs, we explored the possible contribution of WRKY54 and WRKY70 to abiotic stress responses using osmotic stress (PEG6000 treatment) as a model. Our results show that *wrky54wrky70* double mutants present a clearly enhanced tolerance to osmotic stress with reduced stress damage and ion leakage (Fig. 2), in contrast with wild-type plants and single mutants, suggesting the co-operation of these two TFs as negative regulators of the osmotic stress response. Interestingly, as already observed for senescence (Besseau *et al.*, 2012), WRKY70 seems to be more efficient than WRKY54 in this regulatory process. Indeed, in experiments performed with single and double mutants (Figs7), we often observed intermediate phenotypes in the *wrky70* single mutant relative to the *wrky54wrky70* and wild-type, whereas weak or no phenotypic differences were observed between *wrky54* and wild-type plants. In conclusion, our results demonstrate that WRKY54 and WRKY70 modulate abiotic stress tolerance in plants, and indicate that these two TFs co-operate as negative regulators of osmotic stress tolerance (Fig. 10), with WRKY70 playing a more prominent role in this regulation.

**Figure:**
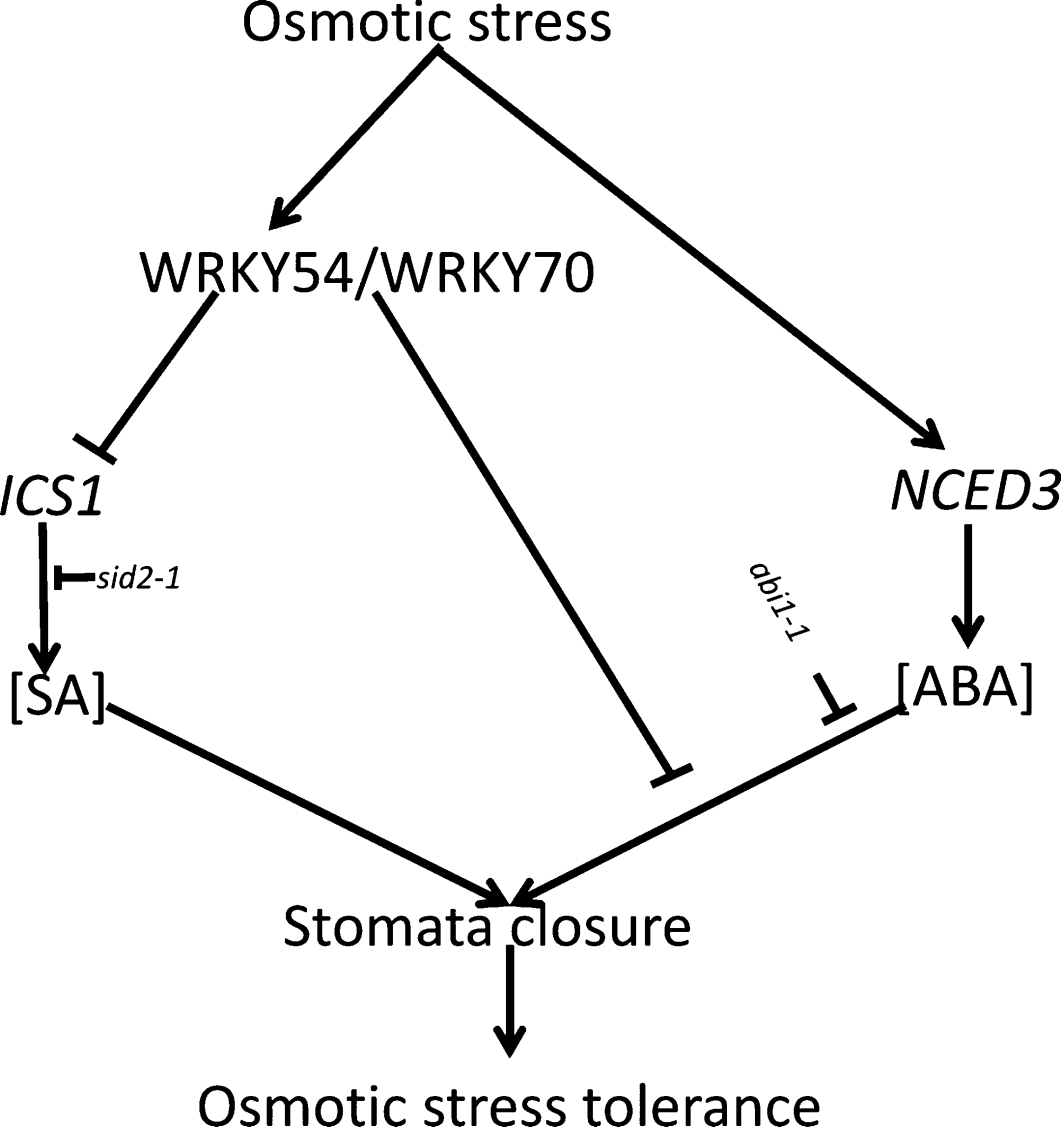
A schematic model of WRKY54- and WRKY70-mediated control of osmotic stress tolerance in Arabidopsis. WRKY54 and WRKY70 negatively modulate the osmotic stress tolerance through the control of stomatal closure, rather than the expression of stress-inducible genes. The arrows indicate induction or positive modulation; the blunt-end arrows represent block or suppression. ABA, abscisic acid; SA, salicylic acid.

### Osmotic stress-induced gene expression is suppressed in the *wrky54wrky70* double mutant as a result of the accumulation of SA

The observed increase in osmotic tolerance of *wrky54wrky70* plants was not explained by the enhanced induction of osmotic stress response genes or enhanced accumulation of protective osmolytes. Indeed, the microarray data (Table 1) showed that the osmotic induction of most of the abiotic stress-responsive genes was partially suppressed in the *wrky54wrky70* double mutant. This suppression was confirmed by qRT-PCR analysis of selected osmotic stress-responsive genes (Fig. 3). Similarly, the accumulation of the osmoprotectant proline was reduced in the double mutant (Fig. 4). We hypothesized that one explanation for this observed suppression could be the increased SA level in the *wrky54wrky70* double mutant (Wang *et al.*, 2006). Indeed, the endogenous levels of free SA and SAG were elevated in the *wrky54wrky70* double mutant under both unstressed and osmotically stressed conditions (Fig. 5). To verify our hypothesis, we introduced the *sid2-1* allele, preventing SA biosynthesis, into the *wrky54wrky70* double mutant. This introduction partially abolished the observed suppression (Table S3). These results were further supported by data showing that exogenous SA also leads to the suppression of osmotically induced expression of abiotic stress-responsive genes (Fig. S2).

What is the mechanism of the suppression by SA? Part of the explanation could lie in the mutual antagonism of SA- and ABA-mediated signaling (Yasuda *et al.*, 2008). ABA is a central component in the abiotic stress response, and its biosynthesis and accumulation are enhanced by drought, salt and cold stress (Xiong *et al.*, 2002). Both our microarray (Table 1) and qRT-PCR (Fig. 3) data showed that the expression of the *NCED3* gene encoding a key enzyme in ABA biosynthesis (Iuchi *et al.*, 2001) was reduced in the *wrky54wrky70* double mutant, suggesting impaired ABA accumulation, and consequently could result in the observed down-regulation of ABA target genes. However, this hypothesis was not supported by the determination of ABA levels in control and osmotically stressed wild-type and mutant lines (Fig. S7). Thus, the antagonistic cross-talk between SA and ABA signaling reported previously (Yasuda *et al.*, 2008) does not appear to take place at the ABA level.

Furthermore, our results suggest that the enhanced osmotolerance observed as a result of the inactivation of *WRKY54* and *WRKY70* is not caused by the increased SA levels in the double mutant. Although the expression of abiotic stress-related genes was restored by introduction of the *sid2-1* allele and a concomitant reduction in SA levels, the osmotic stress tolerance exhibited by the *wrky54wrky70* double mutant was not abolished, although a slight reduction in the enhanced tolerance was observed (Fig. 6). This indicates that the tolerance in *wrky54wrky70* cannot be explained by the increased SA levels, but is a more direct effect of the lack of the negative regulators of osmotolerance, WRKY54 and WRKY70.

### WRKY54 and WRKY70 negatively regulate stomatal closure and this regulation is SA independent

As discussed, the osmotic stress tolerance exhibited by the *wrky54wrky70* double mutant is not explained by the altered expression of abiotic stress-related genes or by the accumulation of osmoprotectants. Rather, it appears that this tolerance phenotype is more directly linked to the control of the plant water balance, as suggested by the clearly reduced water loss and stomatal conductance in the mutant plants (Fig. 7). The results show reduced stomatal conductance in *wrky54wrky70* double mutants relative to the other lines in both unstressed and osmotically stressed plants. These data, supported by the enhanced stomatal conductance in *WRKY70* overexpressors, suggest that WRKY54 and WRKY70 co-operate as negative regulators of stomatal closure (Fig. 10). Part of this regulation could be realized through the control of ABA levels, as suggested by the reduced ABA content in *WRKY70* overexpressors, manifested in the more open stomates and reduced stomatal closure on stress. During osmotic stress, the ABA-mediated signaling pathway is a central element leading to stomatal closure and reduced water loss. Our results suggest that, in this process, osmotic induction of *WRKY54* and *WRKY70* appears to provide a negative feedback loop controlling the stomatal aperture (Fig. 10).

In addition, SA is known to be involved in the control of stomatal movement. For example, in *NahG* and *eds16-2* mutant plants (both deficient in SA), stomatal closure is repressed (Melotto *et al.*, 2008), and a recent report has shown that SA triggers stomatal closure through the production of reactive oxygen species (ROS) and NO in *Arabidopsis* (Khokon *et al.*, 2011). Our data are in accordance with this; stomatal conductance was clearly increased in the SA-deficient *sid2-1* mutant, as well as by introduction of the *sid2-1* allele into the *wrky54wrky70* double mutant, resulting in conductance nearly similar to that of the *sid2-1* mutant itself. However, the osmotically induced stomatal closure in the triple mutant (*wrky54wrky70sid2-*1) was still enhanced (Fig. 7c). These results confirm the positive effect of SA on stomatal closure in agreement with previous reports (Melotto *et al.*, 2008; Acharya & Assmann, 2009; Khokon *et al.*, 2011). However, the results show that the SA over-accumulation in *wrky54wrky70* plants is not responsible for the enhanced stomatal closure observed in this mutant (Fig. 10). By contrast, our data suggest that WRK54 and WRKY70 co-operate as negative regulators of stomatal closure through two pathways: as negative regulators of SA biosynthesis, they keep SA levels down and consequently prevent SA-induced stomatal closure; they have a more direct and SA-independent negative effect on stomatal closure by reducing ABA levels (Fig. 10).

### WRKY54 and WRKY70 control early responses to osmotic stress

ABA is the central hormone mediating drought responses and stomatal movement. The generation of the triple mutant *wrky54wrky70abi1-1* showed that the osmotic stress tolerance of *wrky54wrky70* was abolished by the introduction of the dominant negative *abi1-1* allele (Fig. 9). Interestingly, other WRKY TFs in both rice and *Arabidopsis* have been reported to participate in abiotic stress responses and ABA signaling (Xie *et al.*, 2005; Jiang & Yu, 2009; Chen *et al.*, 2010; Ren *et al.*, 2010; Shang *et al.*, 2010) and, as shown here, are induced by osmotic stress (Fig. S1). In contrast with our work, they seem to act mostly as positive regulators of stress tolerance, although conflicting results have been obtained (Chen *et al.*, 2010; Shang *et al.*, 2010). WRKYs bind to the W-box sequence in promoters of downstream genes and, indeed, some ABA signaling-related genes contain such sequences (Ren *et al.*, 2010; Shang *et al.*, 2010). WRKY40, for example, binds to promoters of *ABI4*, *ABI5* and *ABF4* or other ABA-responsive genes, modulating their expression (Shang *et al.*, 2010); WRKY63 has been shown to bind to the promoter of *ABF2*, positively regulating *ABF2* expression and promoting ABA-mediated stomatal closure. The decreased stomatal conductance in the *wrky54wrky70* background and corresponding microarray data (Tables1, S3, S4) suggested that WRKY54 and WRKY70 might work as negative regulators of an early step of the plant response to osmotic stress, that is, regulation of the stomatal aperture. This notion is supported by their rapid, but transient, induction by osmotic stress and the effect of these WRKYs on the rapid regulation of the stomatal aperture (Fig. 8). By contrast, they are not involved in the later processes of osmotic adaptation when plants activate their defense system to protect them from injury, including the expression of osmotic stress-related genes or the accumulation of osmoprotectants (Verslues *et al.*, 2006; Ramírez *et al.*, 2009). As shown in Fig. 7, the reduced stomatal conductance was evident in the *wrky54wrky70* double mutant before exposure to osmotic stress, and was further reduced by stress, which indicated the role of WRKY54 and WRKY70 in the beginning of ABA-controlled stomatal closure. Taken together, WRKY54 and WRKY70 might negatively regulate the early steps of the stomatal closure, but not the later stages of ABA signaling and stress-induced gene expression.

The schematic model presented in Fig. 10 summarizes the involvement of WRKY70 and WRKY54 in osmotic stress responses. Osmotic stress triggers ABA-dependent stomatal closure, as well as the expression of abiotic stress-responsive genes, resulting in increased stress tolerance. *WRKY54* and *WRKY70* are similarly induced by osmotic stress to modulate these processes, acting as negative regulators of stomatal closure, possibly through the control of ABA levels. As these WRKYs also act as negative regulators of SA biosynthesis, and SA has a positive function in stomatal closure, this provides an indirect negative effect on stomatal closure (Fig. 10).

*WRKY54* and *WRKY70* are also responsive to biotic stress and play an important role as positive regulators of plant defense. Consequently, SA-mediated biotic and ABA-mediated abiotic signaling pathways involving WRKY54 and WRKY70 appear to be parallel, but antagonistically related. Similar findings have been reported in rice and grapevine (Qiu & Yu, 2009; Liu *et al.*, 2011; Peng *et al.*, 2011). Consequently, studies on the mode of action of TFs, such as WRKYs, controlling multiple pathways and mediating cross-talk between biotic and abiotic stress responses will be central to our understanding of the stress response priorities in plants, and may have very significant practical applications in plant production.

## Supporting Information

Fig S1 Expression of representative *WRKY* genes in the wild-type in response to osmotic stress. **Fig. S2** Expression level of osmotic stress-responsive genes in the wild-type plant after 15% polyethylene glycol (PEG) treatment in combination with various amounts of salicylic acid (SA). **Fig. S3** Expression of *WRKY54* and *WRKY70* in the wild-type in response to high salt, drought, cold and exogenous abscisic acid (ABA). **Fig. S4** Comparison of salt stress tolerance in Col-WT (wild-type), *wrky54wrky70* and *wrky54wrky70sid2-1* mutants, and the *WRKY70*-overexpressing line (S55). **Fig. S5** Comparison of drought stress tolerance in Col-WT (wild-type), *wrky54wrky70* and *wrky54wrky70sid2-1* mutants, and the *WRKY70*-overexpressing line (S55). **Fig. S6** Comparison of the stomatal density in the wild-type and *wrky54wrky70* double mutant. **Fig. S7** Abscisic acid (ABA) levels in the wild-type (Col-WT), *wrky54wrky70* and *wrky54wrky70sid2-1* mutants, and the *WRKY70*-overexpressing line (S55) exposed to drought stress for 2 h. **Table S2** Primers used for quantitative reverse transcription-polymerase chain reaction (qRT-PCR)

Table S1 Analysis of gene ontology (GO) terms for the genes picked from the normalized dataset of the microarray **Table S3** Comparison of osmotic stress-related gene expression in wild-type plants (Col-WT), *wrky54wrky70*, *sid2-1* and *wrky54wrky70sid2-1* mutants under 15% polyethylene glycol (PEG) treatment during 1 d **Table S4** Genes downregulated by osmotic stress in the wild-type compared with those in the *wrky54wrky70* double mutant
