# Supplementary material for: Defense-related transcription factors WRKY70 and WRKY54 modulate osmotic stress tolerance by regulating stomatal aperture in Arabidopsis
Source: New Phytol. 2013 Jul 1;200(2):457–72. doi: 10.1111/nph.12378 (PMC4284015; doi:10.1111/nph.12378)
Supplement: Fig S1 — Expression of representative WRKY genes in the wild-type in response to osmotic stress. Fig. S2 Expression level of osmotic stress-responsive genes in the wild-type plant after 15% polyethylene glycol (PEG) treatment in combination with various amounts of salicylic acid (SA). Fig. S3 Expression of WRKY54 and WRKY70 in the wild-type in response to high salt, drought, cold and exogenous abscisic acid (ABA). Fig. S4 Comparison of salt stress tolerance in Col-WT (wild-type), wrky54wrky70 and wrky54wrky70sid2-1 mutants, and the WRKY70-overexpressing line (S55). Fig. S5 Comparison of drought stress tolerance in Col-WT (wild-type), wrky54wrky70 and wrky54wrky70sid2-1 mutants, and the WRKY70-overexpressing line (S55). Fig. S6 Comparison of the stomatal density in the wild-type and wrky54wrky70 double mutant. Fig. S7 Abscisic acid (ABA) levels in the wild-type (Col-WT), wrky54wrky70 and wrky54wrky70sid2-1 mutants, and the WRKY70-overexpressing line (S55) exposed to drought stress for 2 h. Table S2 Primers used for quantitative reverse transcription-polymerase chain reaction (qRT-PCR) [file nph0200-0457-SD1.docx]

**Supporting Information Fig. S1–S7 and Table S2**


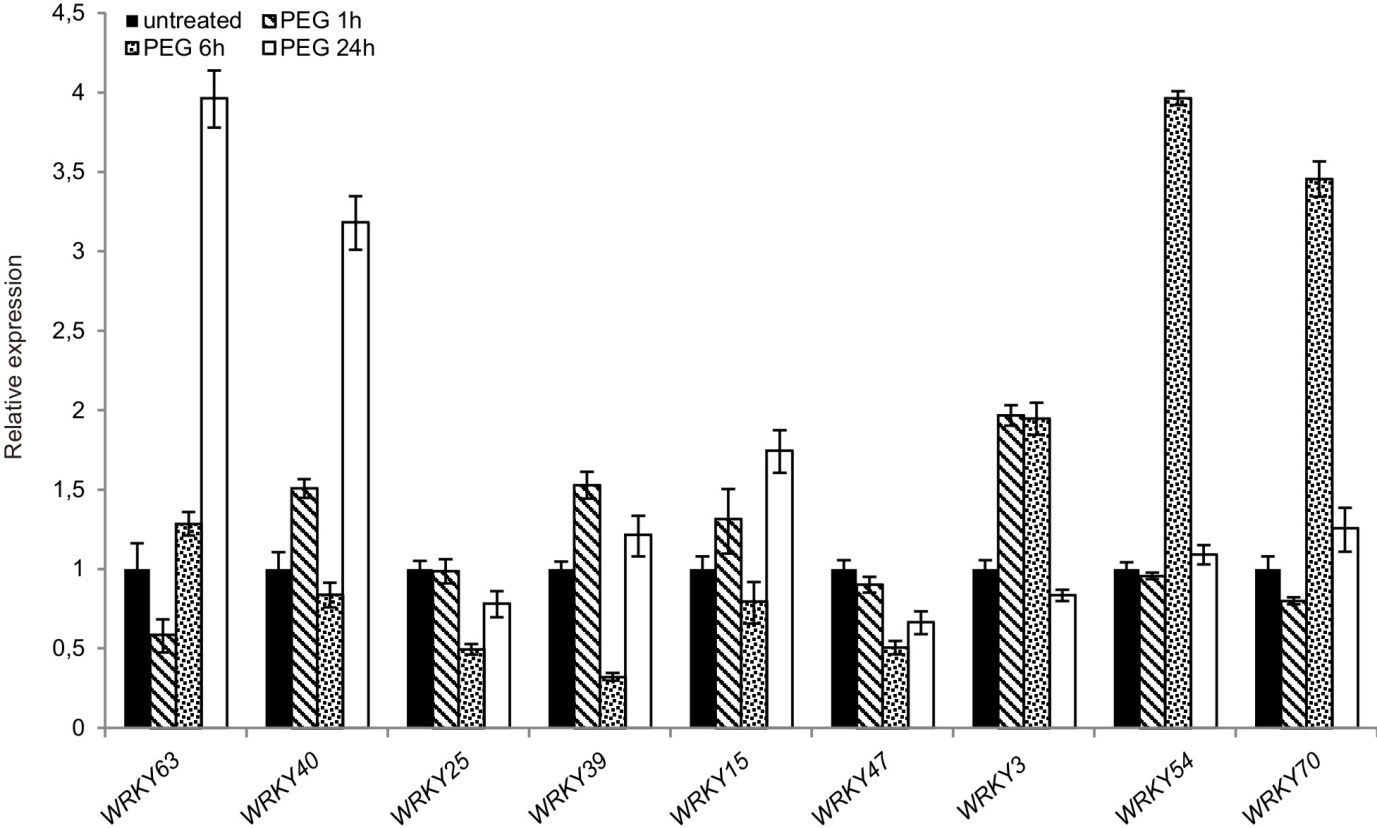


**Fig. S1**

**Fig. S1** Expression of representative *WRKY* genes in wild type in response to osmotic stress. Total RNAs were isolated from 3-wk-old plants watered with 15% PEG solution at indicated time points and gene expression was analyzed by RT-qPCR. The wild type treated with mock solution (water) was used as control. Values represent the means of three replicates. Three independent assays were performed with the similar results.


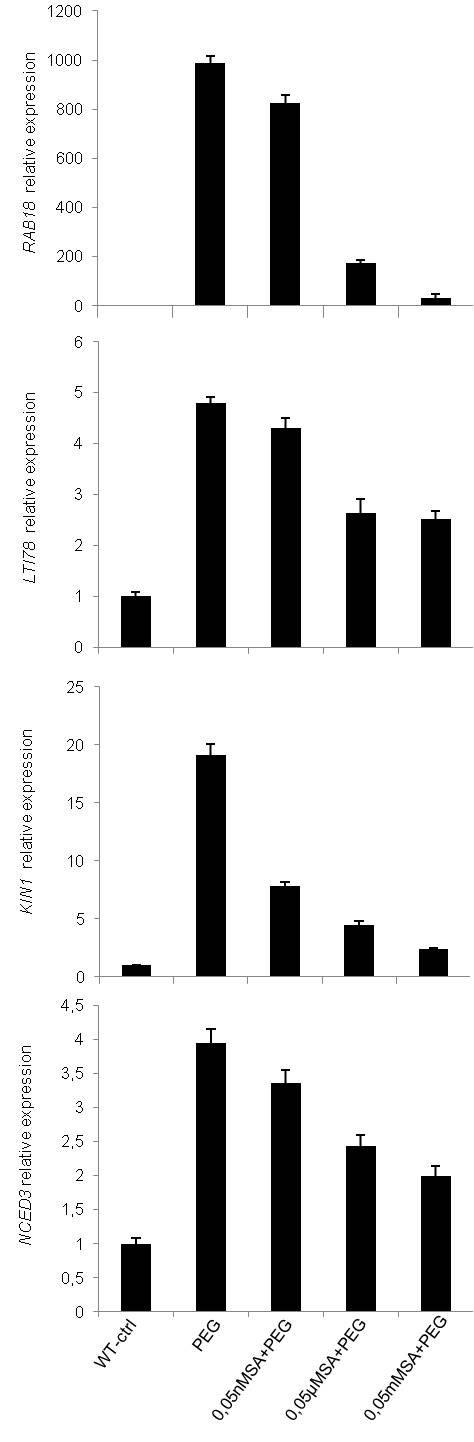


**Fig. S2**

**Fig. S2** Expression level of osmotic stress responsive genes in wild type plant after 15% PEG treatment in combination with various SA amount. Expression measurement of *RAB18*, *LTI78*, *KIN1* and *NCED3* were performed by qRT-PCR on 3-wk-old wild-type plants, 1 d after treatment. The relative expression of each gene was normalized to *ACT2* gene. Values were obtained from three independent experiments. Error bars indicate ±SD.


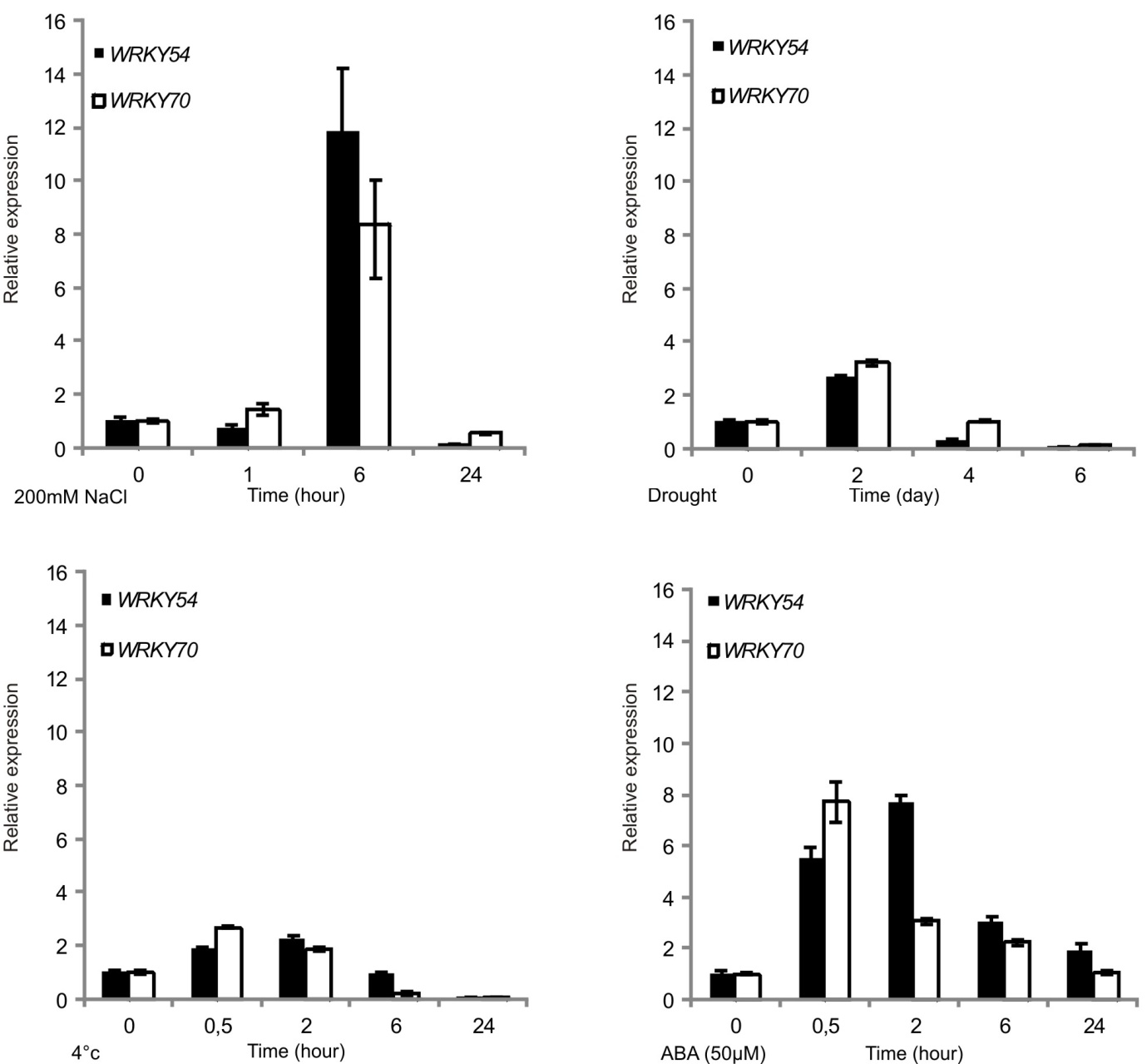


**Fig. S3**

**Fig. S3** The expression of *WRKY54* and *WRKY70* in wild type in response to high salt, drought, cold and exogenous ABA. Total RNAs were isolated from 3-wk-old plants exposed to different stresses and ABA at indicated time points and gene expression was analyzed by RT-qPCR. The wild type treated with mock solution (water) or without treatment was used as control. Values represent the means of three replicates. Three independent assays were performed with the similar results.

**
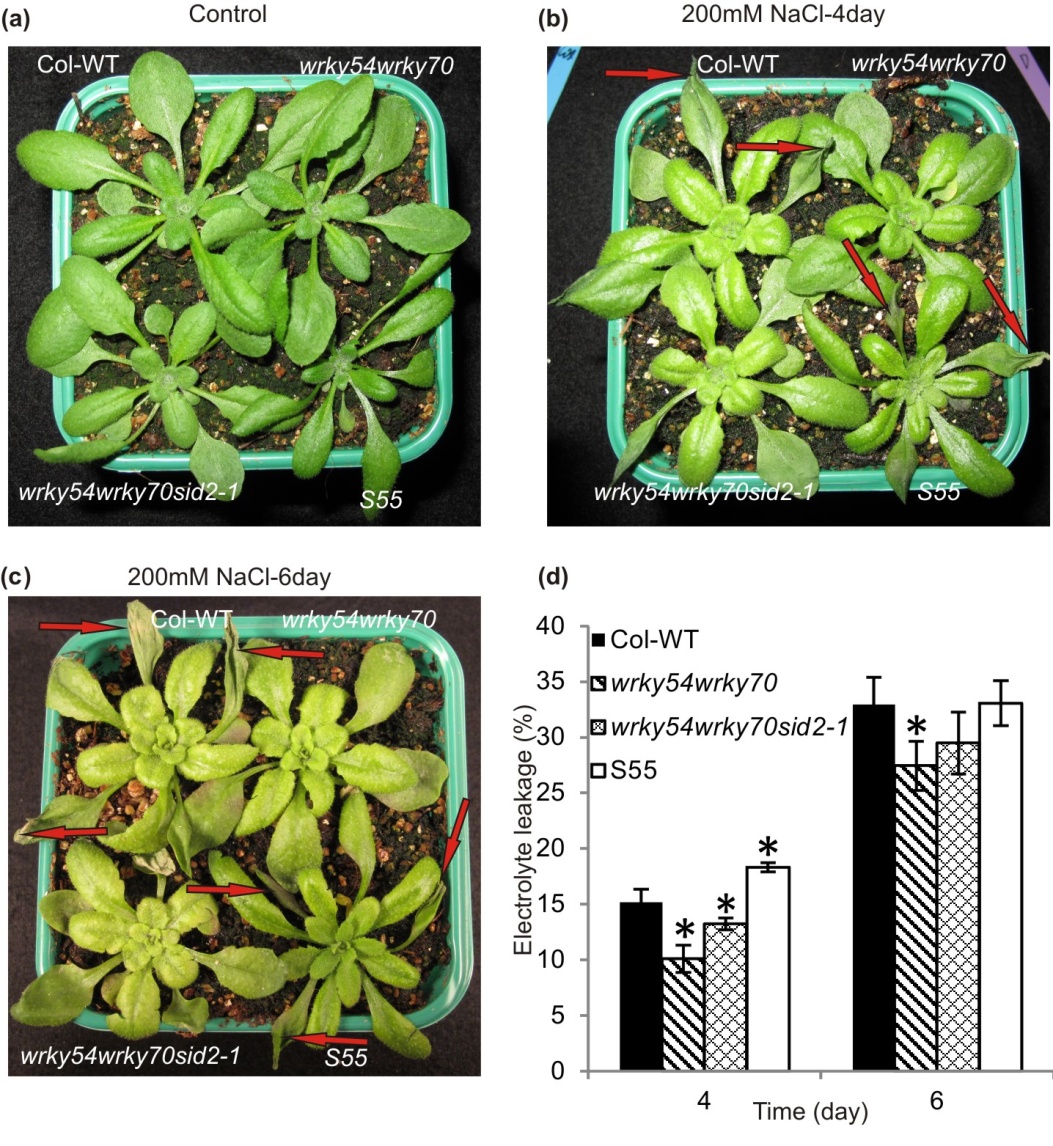
**

**Fig. S4**

**Fig. S4** Comparison of salt stress tolerance in Col-WT, *wrky54wrky70*, *wrky54wrky70sid2-1* mutants and *WRKY70* overexpressing line (S55).

(a,b,c) Three weeks old plants of wild type (Col-WT), *wrky54wrky70,* *wrky54wrky70sid2-1* mutant lines and *WRKY70* overexpressing line (S55) were exposed to salt stress by watering with 200mM NaCl for 1 wk. Each genotype was grown in the same pot to receive equivalent treatment. Eight pots were used at the same time and the pictures were taken 4 and 6 d after treatment. Red arrows indicate the wilting symptoms at the tips and edges of the leaves on the fourth day and subsequently spreading to the whole leaves on the sixth day.

(d) Electrolyte leakage was assessed from leaves after exposure to 200 mM NaCl for 4 and 6 d. Five replicates of each line were used for conductivity measurement. Error bars indicate standard deviations from five replicates (*, *P*<0.01, One-way ANOVA test).

**
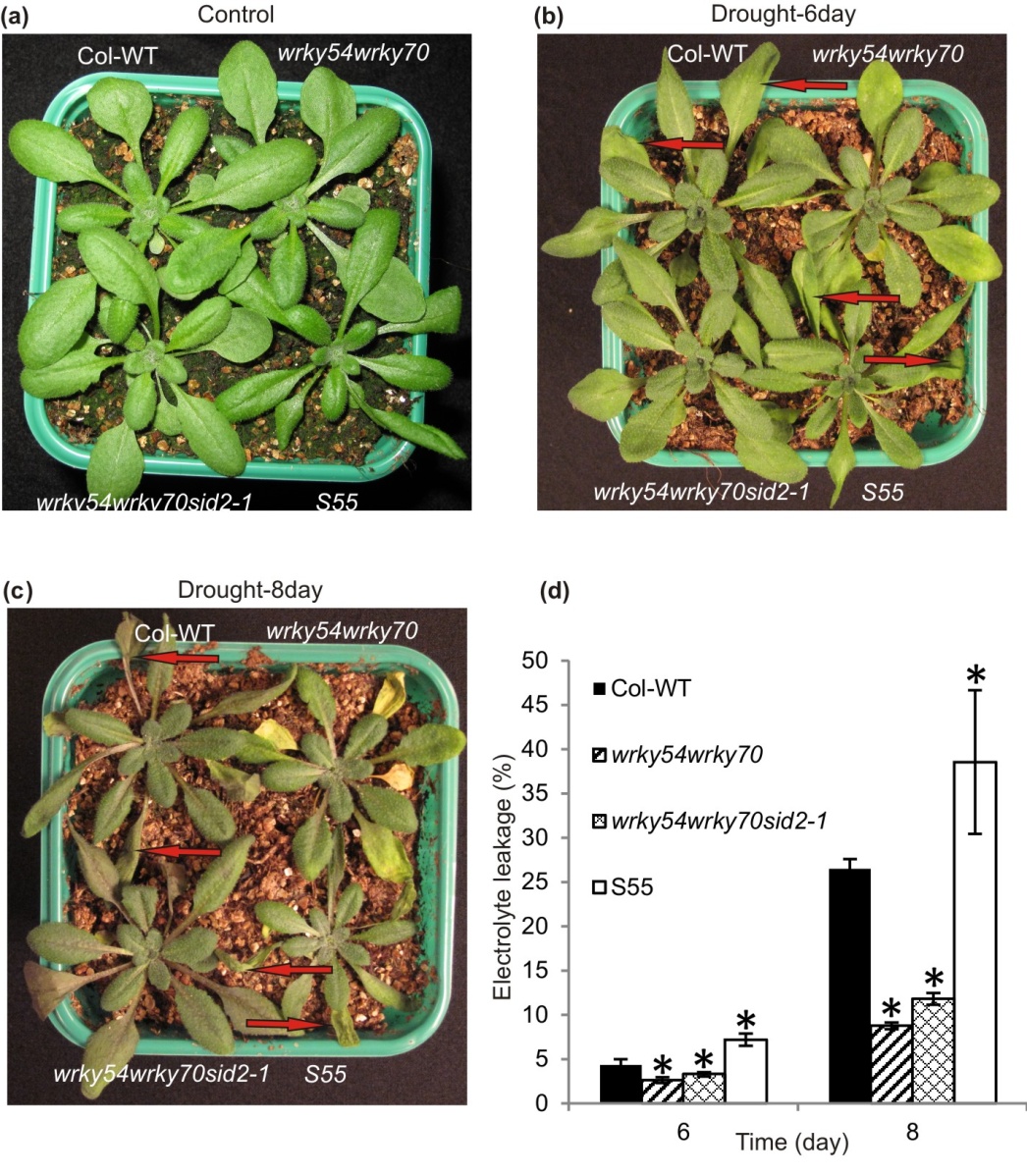
**

**Fig. S5**

**Fig. S5** Comparison of drought stress tolerance in Col-WT, *wrky54wrky70*, *wrky54wrky70sid2-1* mutants and *WRKY70* overexpressing line (S55).

(a,b,c) Three weeks old plants of wild-type plant (Col-WT), *wrky54wrky70,* *wrky54wrky70sid2-1* mutant lines and *WRKY70* overexpressing line (S55) were exposed to drought stress by first watering normally and then the water was withheld for 2 wk. Each genotype was grown in the same pot to receive equivalent treatment. Eighteen pots were used at the same time and the pictures were taken on the sixth and eighth days after water was withheld. Red arrows indicate the wilted leaves in different lines.

(d) Electrolyte leakage was assessed from leaves after exposure to drought stress for 6 and 8 d. Five replicates of each line were used for conductivity measurement. Error bars indicate standard deviations from five replicates (*, *P*<0.01, One-way ANOVA test).


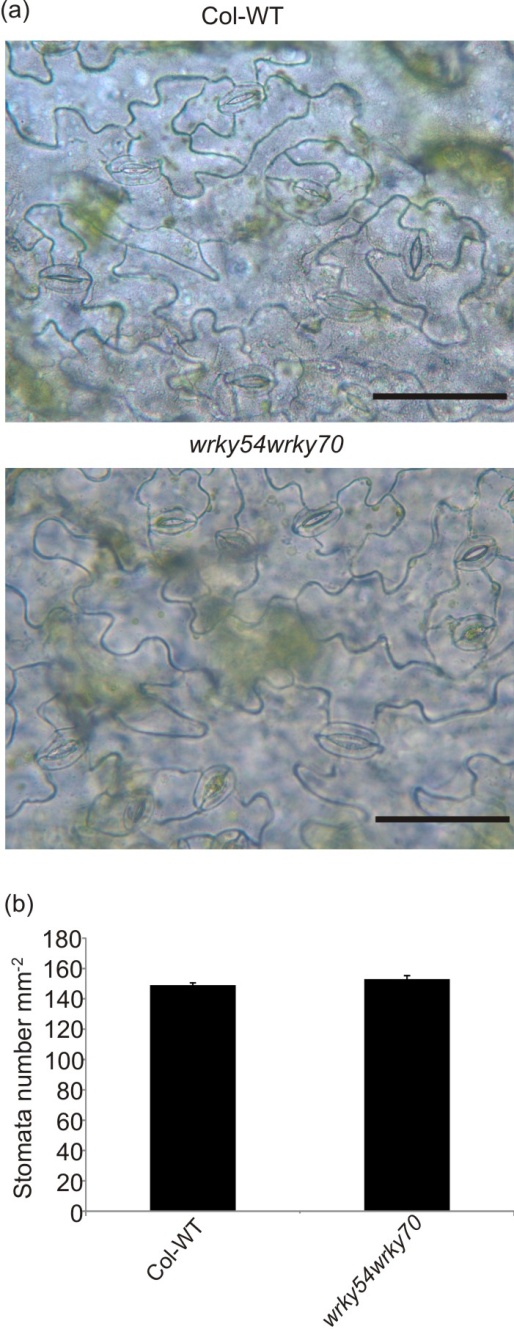


**Fig. S6**

**Fig. S6** Comparison of the stomatal density in wild type and *wrky54wrky70* double mutant.

(a) Comparison of stomatal density between wild type and *wrky54wrky70* double mutant. The density was measured in 0.062 mm^2^ of the leaf area on epidermal peels, scale bar = 50µm. This experiment was repeated three times with the similar results.

(b) Stomatal number per mm^2^ was calculated between wild type and *wrky54wrky70* double mutant, data represent mean values ±SD of three independent experiments conducted with different plant leaves.


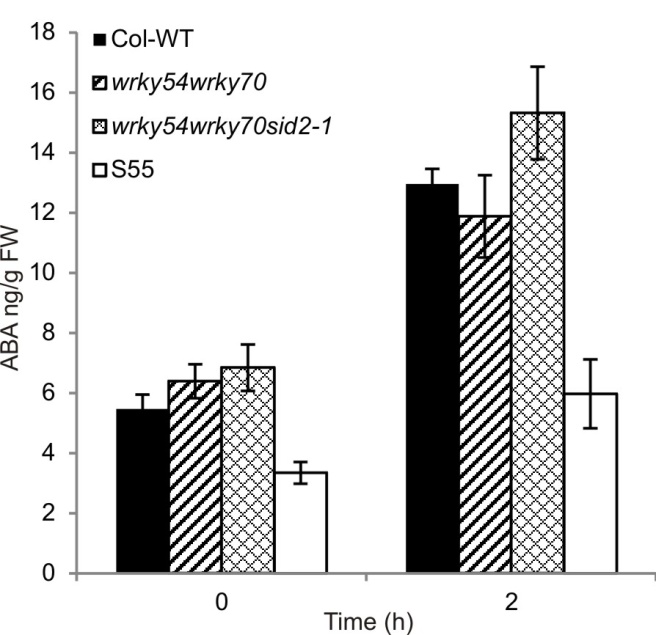


**Figure S7**

**Fig. S7** ABA levels in wild-type (Col-WT), *wrky54wrky70,* *wrky54wrky70sid2-1* mutants and *WRKY70* overexpressing line (S55) exposed to drought stress for 2 h. The content of ABA in leaves of 4-wk-old soil-grown plants was analyzed by UPLC. Analysis was performed on unstressed and drought stressed plants. Values were mean ±SD of five individual replicates of each line.

**Table S2** Primers used for qRT-PCR.

| Gene name | AGI code | Forward primer | Reverse primer |
| --- | --- | --- | --- |
| *WRKY54* | AT2G40750 | GCACTGCTCAGAACCATGTCAA | CAAGTCCTCACCTGTCGAAGA |
| *WRKY70* | AT3G56400 | CATGGATTCCGAAGATCACA | CTGGCCACACCAATGACAA |
| *RAB18* | AT5G66400 | ACGAGTACGGAAATCCGATG | ACCACCACTTTCCTTGTGGA |
| *LTI78* | AT5G52310 | TGGTTGGGAGGATTAAAGGA | AACCAGCCAGATGATTTTGG |
| *KIN1* | AT5G15960 | TGGAGCTGGAGCACAACA | GACCCGAATCGCTACTTGTTC |
| *NCED3* | AT3G14440 | AAAGCCATCGGTGAGCTTCA | GCAGCTCTGGCGTAGAATAGC |
| *P5CS1* | AT2G39800 | TTGTGATCCCAAGAGGAAGC | CGCTTTGCCATATCCGTATC |
| *ProDH* | AT3G30775 | GTTGCAAGTCGCCAGTCCA | GAATCAGCGTTATGTGTTGCGA |
| *ACTIN2* | AT3G18780 | TCAGATGCCCAGAAGTGTGTT | CCGTACAGATCCTTCCTGATAT |
| *WRKY63* | AT1G66600 | CTGTGGCAGCACTCCTTCATGG | CACAAGACCTGCCATGTCTCGAG |
| *WRKY40* | AT1G80840 | AAATCAGCCCTCCCAAGAAACG | CTTCACGACAGTCTCTTCTCTCTG |
| *WRKY25* | AT2G30350 | CCACCTCTTCCGATTTCACA | GTTGTTCCATTAAAGCCTTGC |
| *WRKY39* | AT3G04670 | TGCGGAAGTCGAAGCAAATGTCA | CGTGGATGCGGTGAACCCTTTAT |
| *WRKY15* | AT2G23320 | TCGTTGTCATTGCTCGAAGA | CTTATCGCCGGAACCCTAAT |
| *WRKY47* | AT4G01720 | GCAGCAGCCATGCTCTTATCA | GGTGGGTTGGTGAGGTCTAAGG |
| *WRKY3* | AT2G03340 | GTGAAGGGGAGCGATTTTCC | TGTTACCGCGCTTTTGAGGA |
